# A Novel 1,8-Naphthyridine-2-Carboxamide Derivative Attenuates Inflammatory Responses and Cell Migration in LPS-Treated BV2 Cells via the Suppression of ROS Generation and TLR4/Myd88/NF-κB Signaling Pathway

**DOI:** 10.3390/ijms22052527

**Published:** 2021-03-03

**Authors:** Phuong Linh Nguyen, Bich Phuong Bui, Heesoon Lee, Jungsook Cho

**Affiliations:** 1 Department of Pharmacy, College of Pharmacy and Integrated Research Institute for Drug Development, Dongguk University-Seoul, Goyang, Gyeonggi 10326, Korea; phuonglinh212126@gmail.com (P.L.N.); bichphuong2306@gmail.com (B.P.B.); 2 Department of Pharmaceutics, College of Pharmacy, Chungbuk National University, Osong, Cheongju 28160, Korea

**Keywords:** 1,8-naphthyridine-2-carboxamides, nuclear factor-kappa B, Toll-like receptor, BV2 microglial cells, inflammatory mediators, cell migration, neuroinflammation

## Abstract

Novel 1,8-naphthyridine-2-carboxamide derivatives with various substituents (HSR2101-HSR2113) were synthesized and evaluated for their effects on the production of pro-inflammatory mediators and cell migration in lipopolysaccharide (LPS)-treated BV2 microglial cells. Among the tested compounds, HSR2104 exhibited the most potent inhibitory effects on the LPS-stimulated production of inflammatory mediators, including nitric oxide (NO), tumor necrosis factor-α, and interleukin-6. Therefore, this compound was chosen for further investigation. We found that HSR2104 attenuated levels of inducible NO synthase and cyclooxygenase 2 in LPS-treated BV2 cells. In addition, it markedly suppressed LPS-induced cell migration as well as the generation of intracellular reactive oxygen species (ROS). Moreover, HSR2104 abated the LPS-triggered nuclear translocation of nuclear factor-κB (NF-κB) through inhibition of inhibitor kappa Bα phosphorylation. Furthermore, it reduced the expressions of Toll-like receptor 4 (TLR4) and myeloid differentiation factor 88 (MyD88) in LPS-treated BV2 cells. Similar results were observed with TAK242, a specific inhibitor of TLR4, suggesting that TLR4 is an upstream regulator of NF-κB signaling in BV2 cells. Collectively, our findings demonstrate that HSR2104 exhibits anti-inflammatory and anti-migratory activities in LPS-treated BV2 cells via the suppression of ROS and TLR4/MyD88/NF-κB signaling pathway. Based on our observations, HSR2104 may have a beneficial impact on inflammatory responses and microglial cell migration involved in the pathogenesis of various neurodegenerative disorders.

## 1. Introduction

Microglia, a unique and highly specialized population of brain-resident macrophages, are considered a double-edged sword due to their neuroprotective and neurotoxic effects [1,2]. Microglial cells play essential roles in brain development and are responsible for maintaining central nervous system (CNS) homeostasis [3]. However, upon brain damage or injury, they become activated and their morphology and function undergo a dramatic transformation. Over-activated microglia produce excessive amounts of pro-inflammatory mediators including inflammatory cytokines, tumor necrosis factor-α (TNF-α), nitric oxide (NO), and reactive oxygen species (ROS), which eventually trigger neurodegenerative processes in the brain [1,4,5]. Moreover, microglial stimulation causes rapid changes in their migratory properties to recruit other microglial cells toward the lesion sites [6]. During the last few decades, the hyperactivity of microglia in response to various inflammatory stimuli has been implicated in the onset and development of neuropathies in many neurodegenerative diseases including Alzheimer’s disease (AD), Parkinson’s disease (PD), and multiple sclerosis [7,8,9,10].

Toll-like receptors (TLRs), which are largely expressed on sentinel cells such as macrophages and microglia, participate in the first line of defense against invading pathogens and play important roles in innate immune responses [11,12]. Among the many TLRs identified, TLR4 was shown to be mainly expressed on microglia and to mediate neuroinflammatory responses through the initiation of inflammatory cascades [13,14,15]. Briefly, activated TLR4 recruits an intracellular adaptor protein, myeloid differentiation factor 88 (MyD88), which subsequently triggers several downstream signaling pathways. The major pathway initiated by TLR4-MyD88 interactions includes the nuclear translocation of nuclear factor κB (NF-κB) via the phosphorylation of inhibitor κB (IκB) proteins [16]. The NF-κB translocated to the nucleus promotes the transcription of a series of pro-inflammatory mediators and enzymes associated with inflammatory processes, ultimately leading to neurodegeneration [12,16,17]. In addition, TLR4 activation is known to cause oxidative stress through the enhanced production of intracellular ROS, which further exacerbates inflammatory processes [18]. Therefore, modulation of microglial activation by suppression of the TLR4/MyD88/NF-κB signaling pathway may be a promising strategy to prevent or alleviate neuroinflammation associated with various neurodegenerative brain disorders including AD and PD.

**Figure 1:**
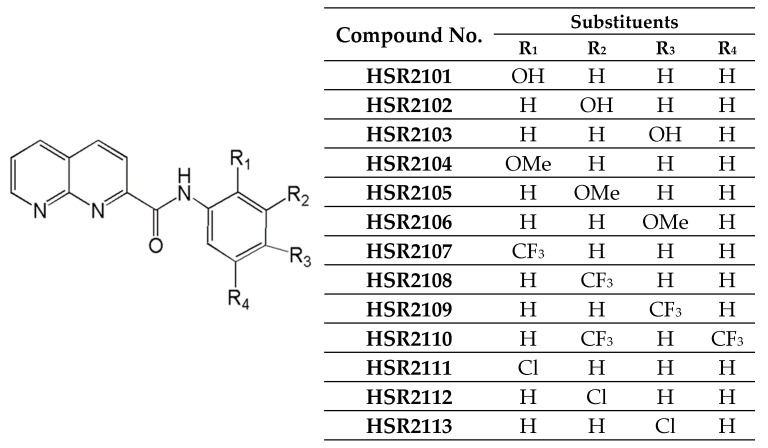
Parent structure of 1,8-naphthyridine-2-carboxamides and their substituents.

Lipopolysaccharide (LPS), one of the best studied immuno-stimulatory endotoxins expressed in the external membrane of Gram-negative bacteria, is commonly used to induce microglial activation [15,19]. LPS is known to trigger neuroinflammatory processes by inducing the release of various cytokines and eicosanoids through the activation of TLR4 [19,20,21]. Using LPS-stimulated BV2 microglial cells, we recently reported the anti-inflammatory and anti-migratory effects of 1,2,3,4-tetrahydroquinoline [22] and isoquinoline-1-carboxamide derivatives [23]. In an effort to seek other agents possessing anti-inflammatory properties, we modified the scaffold of these compounds to naphthyridine to create a series of 1,8-naphthyridine-2-carboxamide derivatives with *N*-hydroxyphenyl, *N*-methoxyphenyl, *N*-(trifluoromethyl)phenyl, *N*-bis(trifluoromethyl)phenyl, or *N*-chlorophenyl substituents (Figure 1).

The present study evaluated the effects of these novel synthetic derivatives on LPS-induced pro-inflammatory mediators and cell migration in BV2 cells. We found that, among the tested compounds, *N*-(2-methoxyphenyl)naphthyridine-2-carboxamide (HSR2104) markedly attenuated the production of pro-inflammatory mediators and cell migration. The signaling pathway involved in the inhibition of inflammatory responses and cell migration was also investigated in this study.

## 2. Results

### 2.1. Effects of 1,8-Naphthyridine-2-Carboxamide Derivatives on the Viability of BV2 Cells

**Figure 2:**
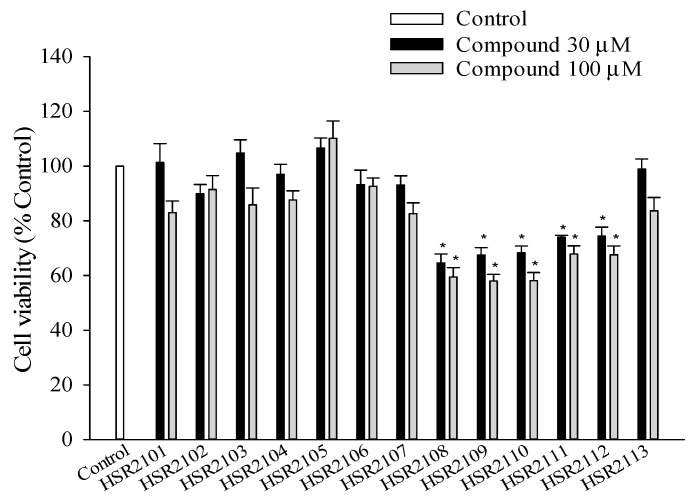
Effects of 1,8-naphthyridine-2-carboxamide derivatives on the viability of BV2 cells. Cells were treated with the compounds at concentrations of 30 µM or 100 µM for 24 h. Control cells were treated with vehicle only. The cell viability was determined by MTT assay, as described in the Materials and Methods section. Data are expressed as the mean ± SEM from three independent experiments performed in duplicate. * *p* < 0.05 vs. vehicle-treated control cells.

We first examined whether the newly synthesized 1,8-naphthyridine-2-carboxamide derivatives had any cytotoxic effect on BV2 cells. The cells were treated with the compounds at two different concentrations (30 μM and 100 μM) for 24 h, and the viability of the cells was assessed by MTT reduction assay. We found that the derivatives with *N*-hydroxylphenyl (HSR2101-2103) and *N*-methoxylphenyl (HSR2104-2106) substituents did not induce significant cytotoxicity in BV2 cells at the concentrations tested (Figure 2). In contrast, the derivatives with an *N*-(trifluoromethyl)phenyl substituent at R2 (HSR2108) or R3 position (HSR2109), *N*-bis(trifluoromethyl)phenyl substituent at R2 and R4 positions (HSR2110), and *N*-chlorophenyl substituent at R1 (HSR2111) or R2 position (HSR2112) induced marked cytotoxicity at both concentrations tested. Interestingly, however, compounds with an *N*-(trifluoromethyl)phenyl substituent at R1 position (HSR2107) and *N*-chlorophenyl substituent at R3 position (HSR2113) induced no significant toxicity (Figure 2).

We also examined the effects of these compounds on the viability of LPS-treated BV2 cells. Similar results were observed for all derivatives, except HSR2113. Although HSR2113 at the concentration of 30 µM did not induce cytotoxicity in LPS-treated BV2 cells, it induced considerable toxicity at 100 µM (data not shown). Based on our findings, seven compounds (HSR2101–HSR2107) that did not show significant cytotoxicity were chosen for further study. HSR2113 was also investigated further because it did not show toxicity in BV2 cells in the absence of LPS.

### 2.2. Effects of 1,8-Naphthyridine-2-Carboxamide Derivatives on the LPS-Stimulated Production of Pro-Inflammatory Mediators in BV2 Cells

**Figure 3:**
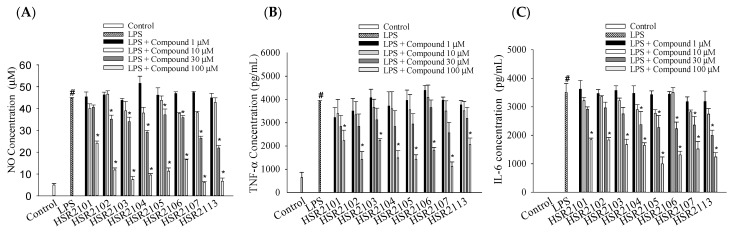
Concentration-dependent inhibition of the lipopolysaccharide (LPS)-stimulated production of pro-inflammatory mediators by the selected 1,8-naphthyridine-2-carboxamide derivatives in BV2 cells. Cells were cotreated with LPS (1 µg/mL) and the indicated concentrations of selected compounds for 24 h. Control cells were treated with vehicle only. Culture media were collected and analyzed for NO (**A**), TNF-α (**B**), and IL-6 (**C**), as described in the Materials and Methods section. Data are expressed as the mean ± SEM from three independent measurements performed in duplicate. # *p* < 0.05 and * *p* < 0.05 vs. vehicle-treated control cells and LPS-treated cells, respectively.

Next, we evaluated the effects of the eight selected compounds (HSR2101–HSR2107 and HSR2113) on the production of LPS-stimulated pro-inflammatory mediators. In agreement with our previous reports [22,23], LPS treatment of BV2 cells dramatically increased the levels of pro-inflammatory molecules, including NO, TNF-α, and IL-6, in the culture media (Figure 3). When the cells were cotreated with LPS and the selected compounds at the concentrations of 1, 10, 30, and 100 µM, all tested compounds exerted significant and concentration-dependent inhibition of the LPS-stimulated production of these inflammatory mediators (Figure 3).

Using the data shown in Figure 3, the IC_50_ values of compounds that inhibited NO, TNF-α, and IL-6 production were calculated (Table 1). Compounds substituted at the ortho position (R1) (HSR2101, HSR2104, HSR2107) exhibited better inhibitory activities against the LPS-stimulated production of TNF-α than those with meta (R2) (HSR2102, HSR2105) or para (R3) substitutions (HSR2103, HSR2106, HSR2113). Of those compounds that induced a marked inhibition of TNF-α production, HSR2104 (*N*-methoxyphenyl substituent) and HSR2107 (*N*-trifluoromethylphenyl substituent) induced a higher inhibition of NO production than HSR2101 (*N*-hydroxyphenyl substituent). Furthermore, IL-6 production in the LPS-treated BV2 cells was markedly inhibited by HSR2104, whereas only mild inhibition was observed by HSR2107. Taken together, HSR2104 (*N*-(2-methoxyphenyl)naphthyridine-2-carboxamide) exhibited the most potent inhibition of the LPS-induced production of NO, TNF-α, and IL-6 in BV2 cells. Therefore, we focused on HSR2104 for further characterization of its anti-inflammatory potential and the underlying mechanism(s) of action. Prior to further characterization of anti-inflammatory properties of HSR2104, we verified that the levels of NO, TNF-α, and IL-6 were not influenced by HSR2104 treatment at 100 µM for 24 h in the absence of LPS).

### 2.3. Effects of HSR2104 on the LPS-Induced iNOS and COX2 Expression in BV2 Cells

**Figure 4:**
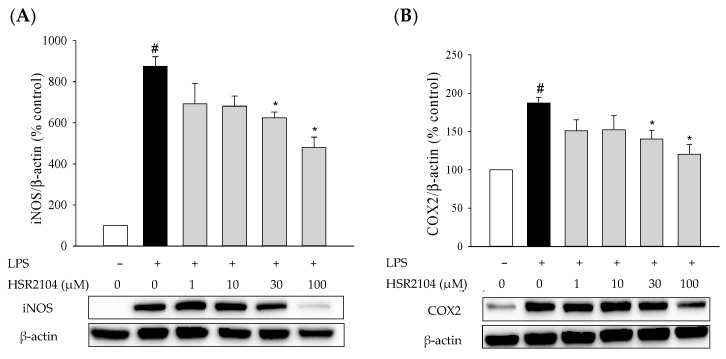
Inhibition of the LPS-induced expressions of iNOS and COX2 by HSR2104 in BV2 cells. Cells were cotreated with LPS (1 µg/mL) and the indicated concentrations of HSR2104 for 24 h. Control cells were treated with vehicle only. The protein levels of iNOS (**A**) and COX2 (**B**) were determined by Western blotting analyses using anti-iNOS and COX2 antibodies, respectively. β-actin was used as an internal control. Representative blots are shown. Data are presented as the mean ± SEM from three independent experiments. # *p* < 0.05 and * *p* < 0.05 vs. vehicle-treated control and LPS-treated cells, respectively.

We then investigated whether the inhibition of the pro-inflammatory mediators by HSR2104 was associated with the modulation of inducible NO synthase (iNOS) and cyclooxygenase 2 (COX2) expressions. Western blotting analyses demonstrated that LPS treatment of BV2 cells markedly enhanced the expressions of iNOS and COX2 (Figure 4), consistent with our previous reports [22,23]. HSR2104 concentration-dependently suppressed the protein levels of LPS-induced iNOS and COX2 (Figure 4). These findings suggest that anti-inflammatory properties of HSR2104 may, at least in part, be attributed to the reduced expressions of iNOS and COX2 in the LPS-treated BV2 cells.

### 2.4. Effect of HSR2104 on the LPS-Induced BV2 Cell Migration

**Figure 5:**
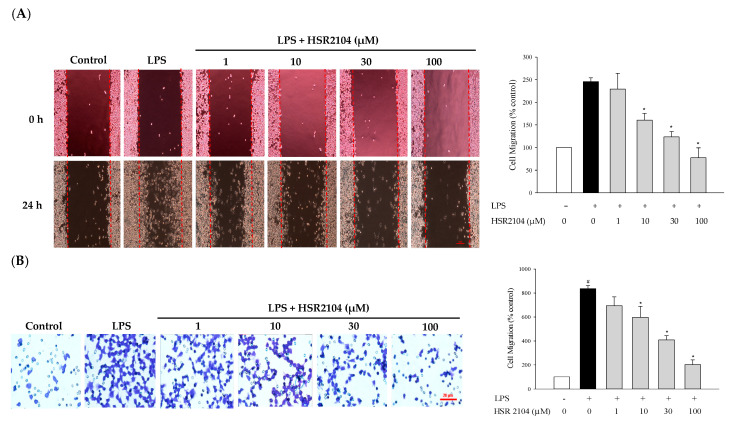
Suppression of LPS-induced BV2 cell migration by HSR2104. Cells were cotreated with LPS (1 µg/mL) and the indicated concentrations of HSR2104 for 24 h and the extent of cell migration was measured by wound healing (**A**) and transwell migration (**B**) assays, as described in the Materials and Methods section. Control cells were treated with vehicle only. Representative microscopic images are shown (scale bar, 20 μm). Data are expressed as the mean ± SEM from at least three independent experiments. # *p* < 0.05 and * *p* < 0.05 vs. vehicle-treated control and LPS-treated cells, respectively.

Microglial cell movement is conjunctly associated with inflammatory responses [6,24]. Therefore, we investigated the effect of HSR2104 on LPS-induced BV2 cell migration using wound healing and transwell migration assays. As described in our previous studies [22,23], LPS dramatically enhanced BV2 cell migration during 24 h of treatment (Figure 5). Our results revealed that the concomitant treatment of cells with LPS and HSR2104 at the concentrations of 10 µM and above markedly repressed LPS-induced cell migration in both assays (Figure 5). Additional wound healing assay showed that the migratory activity of HSR2104-treated cells in the absence of LPS was similar to that of vehicle-treated control cells (data not shown).

### 2.5. Effect of HSR2104 on the LPS-Stimulated Generation of Intracellular ROS in BV2 Cells

**Figure 6:**
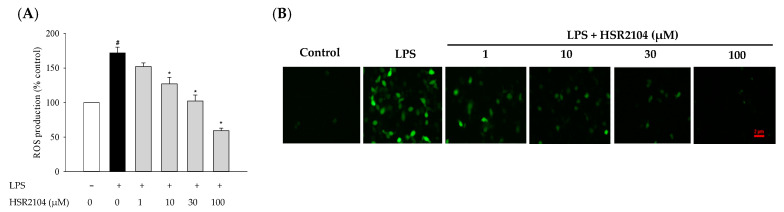
Inhibition of LPS-induced intracellular ROS production by HSR2104 in BV2 cells. Cells were cotreated with LPS (1 µg/mL) and the indicated concentrations of HSR2104 for 24 h. Intracellular ROS levels were determined using DCFH-DA as a fluorescent probe (**A**), as described in the Materials and Methods section. Control cells were treated with vehicle only. Data are expressed as the mean ± SEM from three independent experiments in triplicate. Representative fluorescence microscopic images (**B**) are shown (scale bar, 2 µm). # *p* < 0.05 and * *p* < 0.05 vs. vehicle-treated control and LPS-treated cells, respectively.

The generation of intracellular ROS in microglia is acknowledged to trigger neuroinflammatory responses through the activation of several signaling pathways including the NF-κB pathway [18]. Thus, we examined the effect of HSR2104 on the generation of ROS in LPS-stimulated BV2 cells. The production of intracellular ROS in the cells treated with LPS was significantly increased to approximately 180%, compared to that in the control-treated cells (Figure 6). Cotreatment with LPS and HSR2104 attenuated ROS generation in a concentration-dependent manner (Figure 6A). The inhibition of ROS generation by HSR2104 in LPS-treated BV2 cells was further confirmed by fluorescence microscopic images, which demonstrated a marked reduction of the LPS-stimulated fluorescence signals by HSR2104 (Figure 6B).

### 2.6. Effects of HSR2104 on the LPS-Induced IκBα Phosphorylation and NF-κB Translocation in BV2 Cells

**Figure 7:**
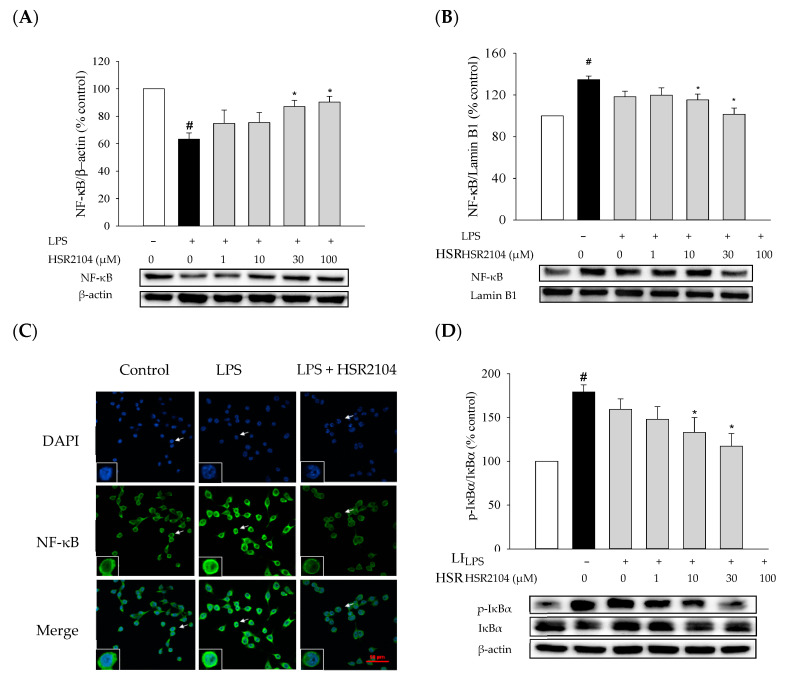
Inhibition of NF-κB nuclear translocation by HSR2104 through the inhibition of IκBα phosphorylation in LPS-treated BV2 cells. Cells were cotreated with LPS (1 µg/mL) and the indicated concentrations of HSR2104 for 24 h. Control cells were treated with vehicle only. Cytosolic (**A**) and nuclear (**B**) fractions were prepared and Western blotting analyses were performed using anti-NF-κB p65 antibodies, as described in the Materials and Methods section. β-actin and lamin B1 were used as internal controls for the cytosolic and nuclear fractions, respectively. Representative blots are shown. (**C**) Cells were cotreated with LPS (1 µg/mL) and HSR2104 at 100 µM for 24 h, and immunocytochemical analyses were performed. The localization of NF-κB was visualized in green using anti-NF-κB p65 antibodies, and the nuclei were visualized in blue with DAPI staining. Representative immunofluorescence images are shown. The white arrow in each image indicates the magnified cell shown in the inset (scale bar, 50 µm). (**D**) Cells were cotreated with LPS (1 µg/mL) and the indicated concentrations of HSR2104 for 24 h. Cell lysates were prepared and analyzed by Western blotting with anti-phospho-IκBα and anti-IκBα antibodies. β-actin was used as an internal control. Representative blots are shown. Data are presented as the mean ± SEM from at least three independent experiments. # *p* < 0.05 and * *p* < 0.05 vs. vehicle-treated control and LPS-treated cells, respectively.

Next, we investigated the involvement of NF-κB pathway in the inhibition of LPS-induced inflammatory responses by HSR2104. As reported previously [22,23], LPS treatment induced nuclear translocation of NF-κB in BV2 cells. Immunoblotting analyses indicated that, upon LPS treatment, the level of NF-κB p65 subunit was reduced in the cytosolic fraction, while it was increased in the nuclear fraction (Figure 7A,B). LPS-induced NF-κB translocation was also manifested in the immunofluorescence images, showing strong fluorescence intensity of NF-κB in the nuclei of LPS-treated cells (Figure 7C). Both immunoblotting and immunocytochemical analyses demonstrated that HSR2104 reversed the LPS-induced NF-κB translocation (Figure 7A–C). The expression of NF-κB p65 subunit was augmented in the cytosolic fraction by HSR2104, whereas it was significantly reduced in the nuclear fraction (Figure 7A,B). Similarly, the fluorescence intensity of NF-κB in the nucleus was markedly diminished by HSR2104 at the concentration of 100 µM (Figure 7C).

It is widely recognized that the nuclear translocation of NF-κB is mediated by phosphorylation of IκBα [15,16]. Thus, we also tested the effect of HSR2104 on the phosphorylation of IκBα. As shown in Figure 7D, HSR2104 abated the LPS-induced phosphorylation of IκBα at the concentrations of 30 µM and above. Collectively, these results indicate that HSR2104 suppresses the nuclear translocation of NF-κB through inhibition of IκBα phosphorylation.

### 2.7. Effects of HSR2104 on the LPS-Induced Expressions of TLR4 and MyD88 in BV2 Cells

**Figure 8:**
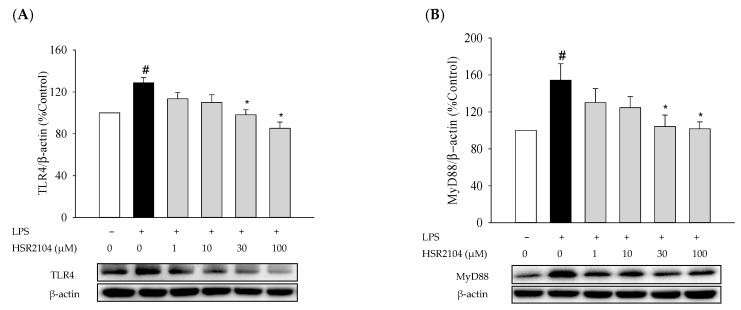
Inhibition of TLR4 and MyD88 expressions by HSR2104 in LPS-treated BV2 cells. Cells were cotreated with LPS (1 µg/mL) and the indicated concentrations of HSR2104 for 24 h. Control cells were treated with vehicle only. The protein levels of TLR4 (**A**) and MyD88 (**B**) were determined by Western blotting analyses using anti-TLR4 and anti-MyD88 antibodies, as described in the Materials and Methods section. β-actin was used as an internal control. Data are expressed as the mean ± SEM from at least three independent experiments. # *p* < 0.05 and * *p* < 0.05 vs. vehicle-treated control and LPS-treated cells, respectively.

The crucial role of the TLR4 pathway in the production of pro-inflammatory molecules has been elucidated in many studies [14,16,23]. In brief, the activation of TLR4 by LPS initiates inflammatory cascades recruiting MyD88, an intracellular adapter protein, and consequently stimulating downstream signaling including the NF-κB pathway. In this study, we observed that HSR2104 inhibited IκBα phosphorylation, and thereby, suppressed the LPS-induced nuclear translocation of NF-κB in BV2 cells (Figure 7). These findings prompted us to investigate the role of TLR4/MyD88 signaling in mediating the anti-inflammatory effects of HSR2104. Immunoblotting analyses revealed that the levels of TLR4 and MyD88 were significantly increased by LPS in BV2 cells, which was reversed by HSR2104 by concentration-dependent fashion (Figure 8). These results suggest that the inhibition of TLR4/MyD88 signaling by HSR2104 may be critically associated with its anti-inflammatory properties.

### 2.8. Effects of TLR4 Inhibitor on the LPS-Induced Pro-Inflammatory Mediators, NF-κB Translocation, Cell Migration, and ROS Production in BV2 Cells

**Figure 9:**
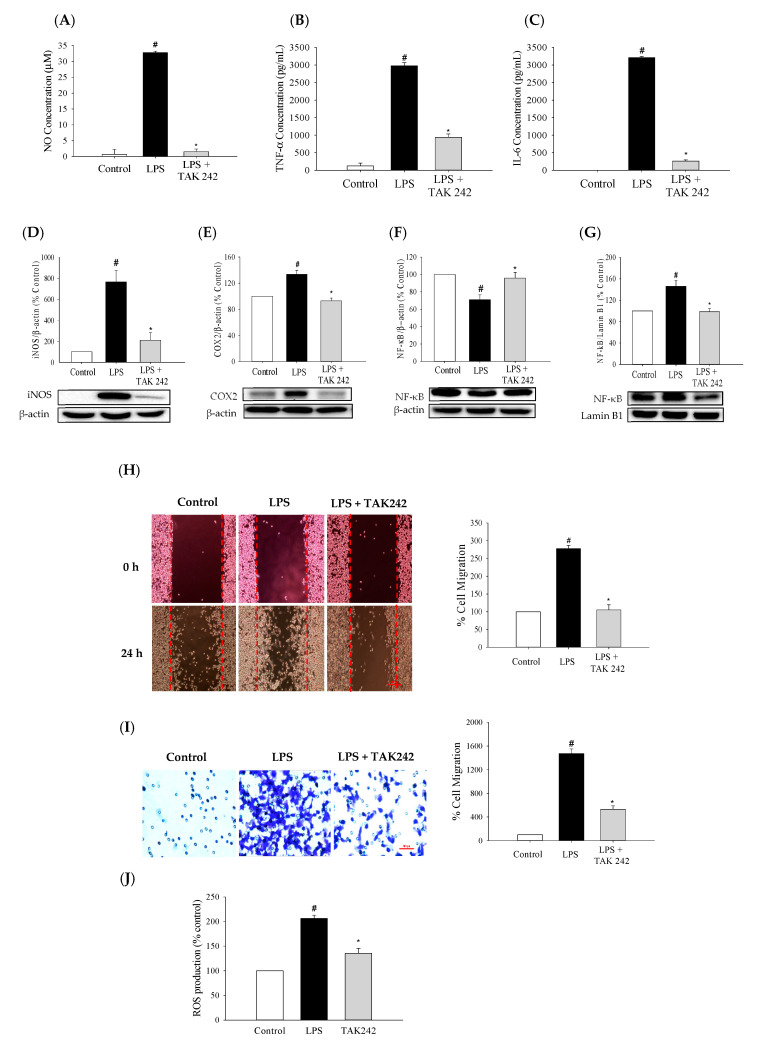
Effects of TAK242, a TLR4-specific inhibitor, on the LPS-induced pro-inflammatory mediators, NF-κB translocation, cell migration, and ROS production in BV2 cells. Cells were cotreated with LPS (1 μg/mL) and TAK242 at the concentration of 500 nM for 24 h. Control cells were treated with vehicle only. Culture media were collected and analyzed for NO (**A**), TNF-α (**B**), and IL-6 (**C**) levels, as described in the Materials and Methods section. Cell lysates were used to perform Western blotting analyses using anti-iNOS (**D**), anti-COX2 (**E**), and anti-NF-κB p65 subunit (**F**,**G**) antibodies. Wound healing (**H**) and transwell migration (**I**) assays were carried out, as described in the Materials and Methods section, to measure cell migration. Intracellular ROS levels were determined using DCFH-DA as a fluorescent probe (**J**). Representative blots and images are shown. Data are expressed as the mean ± SEM from at least three independent experiments. # *p* < 0.05 and * *p* < 0.05 vs. vehicle-treated control and LPS-treated cells, respectively.

The results of the current study demonstrated that HSR2104 inhibited inflammatory responses, cell migration, and ROS generation in LPS-treated BV2 cells. In addition, HSR2104 inhibited the LPS-induced NF-κB translocation and TLR4/MyD88 expressions. To further elucidate the role of TLR4/MyD88 signaling in the anti-inflammatory effects of HSR2104, we investigated the effects of TAK242, a specific inhibitor of TLR4 [25,26], on the LPS-induced pro-inflammatory mediators, NF-κB translocation, cell migration, and ROS production in BV2 cells. We found that TAK242, at the concentration of 500 nM, dramatically inhibited the LPS-induced production of NO, TNF-α, and IL-6 (Figure 9A–C). In addition, LPS-stimulated iNOS and COX2 expressions were markedly diminished by TAK242 (Figure 9D,E). Moreover, TAK242 abolished the LPS-induced nuclear translocation of NF-κB (Figure 9F,G) through the inhibition of IκBα phosphorylation (data not shown). Furthermore, TAK242 suppressed LPS-induced cell migration in the wound healing (Figure 9H) and transwell migration (Figure 9I) assays. The generation of ROS was also quenched by TAK242 (Figure 9J). Collectively, TAK242 and HSR2104 exhibited similar effects on the LPS-induced pro-inflammatory mediators, NF-κB translocation, cell migration, and ROS production in BV2 cells. These findings strongly indicate that TLR4 is an upstream regulator of NF-κB translocation. Moreover, our results confirm that the inhibition of TLR4/MyD88 signaling by HSR2104 plays a crucial role in the inhibition of inflammatory responses, cell migration, and ROS generation in BV2 cells.

## 3. Discussion

It was reported that compounds with naphthyridine scaffolds possess anticancer and anti-inflammatory activities [27,28]. In particular, 1,8-naphthyridine derivatives was demonstrated to exhibit anti-inflammatory effects through the inhibition of ROS production and myeloperoxidase activity in human polymorphonuclear cells [28]. To explore and expand the pharmacological characterization of 1,8-naphthyridine derivatives, the present study synthesized thirteen novel 1,8-naphthyridine-2-carboxamide derivatives and evaluated their anti-inflammatory and anti-migratory activities in BV2 microglial cells.

Microglial activation is closely associated with neuroinflammation, which is involved in the pathologies of various neurodegenerative diseases including AD, PD, and multiple sclerosis [7,8,9,10]. LPS can activate microglial cells and trigger the secretion of various cytokines and eicosanoids, which subsequently promote neuroinflammatory processes [19,20]. BV2 cells, a murine-derived immortalized microglial cell line, are reported to retain many of functional characteristics of primary microglia including responses to LPS [29,30,31]. Upon LPS stimulation, BV2 cells produce various kinds of neuroinflammatory mediators that are deleterious to neurons, eventually leading to progressive neurodegeneration and death [32,33]. Therefore, BV2 microglial cells are widely used as a substituted model for primary microglia in many experimental settings to characterize the cellular and molecular pathways associated with neuroinflammation [15,19,22,23]. The present study also employed LPS-treated BV2 cells to evaluate the anti-inflammatory properties of newly synthesized 1,8-naphthyridine-2-carboxamide derivatives with *N*-hydroxyphenyl, *N*-methoxyphenyl, *N*-(trifluoromethyl)phenyl, *N*-bis(trifluoromethyl)phenyl, or *N*-chlorophenyl substituents (HSR2101-HSR2113) (Figure 1). Prior to evaluation, we first tested their potential cytotoxicity against BV2 cells. Among thirteen compounds tested, eight (HSR2101–HSR2107 and HSR2113) did not exhibit significant cytotoxicity at concentrations up to 100 µM; therefore, these were selected for further investigation (Figure 2).

Neuroinflammation is initiated and exacerbated by diverse pro-inflammatory mediators secreted from activated microglia. For example, NO functions as a chemoattractant directing microglial movement toward lesion sites, where it is produced at a high concentration [6,24]. This leads to the accumulation of activated microglial cells at sites of injury, further promoting their chronic activation [6,34]. In addition, TNF-α and IL-6 are the two main cytokines associated with inflammatory responses. Their abnormal release also plays important roles in microglial activation, neuroinflammation, and leukocyte infiltration to the lesion sites [35,36]. Therefore, we tested the effects of the selected compounds on the LPS-stimulated production of these pro-inflammatory mediators in BV2 cells. All selected compounds significantly inhibited the LPS-induced production of NO, TNF-α, and IL-6 in concentration-dependent manners (Figure 3). Based on their IC_50_ values (Table 1), HSR2104 was chosen for further characterization related to its anti-inflammatory potential and the underlying mechanism(s) of action. Recently, we reported the potent anti-inflammatory effect of an isoquinoline-1-carboxamide derivative with a hydroxyl moiety at the R1 position [23]. In the current study, HSR2104, a 1,8-naphthyridine-2-carboxamide derivative with an *N*-methoxyphenyl substituent at the R1 position (Figure 1), exhibited the most potent inhibition of the LPS-induced production of NO, TNF-α, and IL-6 in BV2 cells (Figure 3, Table 1). These findings imply that the nature and position of substituents may be important in determining the anti-inflammatory properties of these derivatives.

Early studies confirmed the existence of iNOS in microglia, which synthesizes high levels of NO after exposure to stimulant factors such as LPS and viral infections [37,38]. In addition, COX2 was identified as a key enzyme involved in inflammatory responses due to its ability to synthesize various kinds of prostaglandins [39]. The sustained up-regulation of iNOS and COX2 expression in microglia are involved in the increased production of NO and pro-inflammatory cytokines, which exacerbates neuroinflammation and progressive neuronal damage in many neurodegenerative conditions. Therefore, the reduction of iNOS and COX2 expression in microglia might be a potential target for the alleviation of neuroinflammation [40,41]. Our data revealed that HSR2104 markedly inhibited the LPS-induced expressions of iNOS and COX2 (Figure 4), which might contribute to the decreased levels of NO, TNF-α, and IL-6 observed in LPS-treated BV2 cells (Figure 3 and Table 1).

It is well-established that microglia are highly motile, the main characteristic of immune cells [6]. In response to inflammatory stimuli or injury, resting microglial cells are activated by various factors including NO released from damaged cells, leading to their migration towards an infected site [24]. LPS induces the rapid extension of membrane processes and migration of microglia [42], which is in accordance with our data using wound healing and transwell migration assays (Figure 5). Indeed, LPS-induced cell migration was markedly suppressed by HSR2104 (Figure 5).

Oxidative stress resulting from either decreased anti-oxidant systems, increased ROS production, or both, plays a crucial role in the development of various degenerative diseases [43]. It was previously demonstrated that intracellular ROS, including superoxide anions (O2–), hydroxyl radical (·OH), and other types of oxygen-derived radicals, contribute to the etiology of inflammatory processes through the secretion of various inflammatory cytokines [44]. Interestingly, TLR4 signaling mediates the generation of ROS in LPS-stimulated microglia, leading to accelerated inflammatory responses [45,46]. Therefore, the pharmacological blockade of ROS generation may abolish LPS-induced inflammatory responses [47,48,49]. In keeping with previous reports [45,49,50], our study also revealed a marked increase in ROS production in LPS-treated microglia, which was effectively decreased by HSR2104 (Figure 6). Taken together, our findings demonstrate that HSR2104 inhibits LPS-induced inflammatory responses and cell migration by suppressing pro-inflammatory mediators through the inhibition of iNOS and COX2 expressions as well as ROS generation in BV2 cells.

It is well-established that NF-κB plays a predominant role in the regulation of pro-inflammatory cytokines [51,52,53]. NF-κB exists in the cytoplasm in an inactive form where it forms a complex with regulatory IκB proteins. IκBα is associated with the inactive NF-κB heterodimer consisting of p65 and p50 proteins. Phosphorylation of IκBα by IκB kinase (IKK) triggers its dissociation from the cytoplasmic NF-κB-IκB complex allowing it to be targeted for degradation. As a result, activated NF-κB translocates to the nucleus and induces the transcription of a series of genes involved in cell survival or apoptosis, immune responses, and inflammation [53,54,55]. In order to reveal the involvement of NF-κB in the anti-inflammatory and anti-migratory effects of HSR2104, we tested its effects on the nuclear translocation of NF-κB and phosphorylation of IκBα in LPS-treated BV2 cells. Consistent with previous reports [15,22,23,42], LPS treatment promoted the nuclear translocation of NF-κB and enhanced IκBα phosphorylation, which was markedly inhibited by HSR2104 (Figure 7). Our findings indicate that inhibition of the NF-κB pathway also mediates the anti-inflammatory and anti-migratory effects of HSR2104.

The innate immune system is composed of a series of encoded pattern recognition receptors that detect conserved pathogen-associated molecular patterns expressed on microorganisms [56]. TLRs are a type of pattern recognition receptors that play key roles in the innate immune system. Among TLRs, TLR4 expressed on microglial cell membranes is known to recognize LPS as a ligand [12]. Upon binding to LPS, it recruits an adapter molecule MyD88 which subsequently triggers the activation of downstream kinases such as IRAK-4 and TAK1 [12,57]. Activated TAK1 then phosphorylates IκBα via the activation of IKK, which eventually promotes NF-κB nuclear translocation and inflammatory cytokine production [57]. The present study examined the effect of HSR2104 on LPS-activated TLR4/MyD88 signaling in BV2 cells to clarify the involvement of this signaling pathway in its anti-inflammatory and anti-migratory effects. We found that LPS treatment markedly increased the expressions of TLR4 and MyD88 in BV2 cells, which was dramatically suppressed by HSR2104 (Figure 8). These results suggest that HSR2104 exerts anti-inflammatory and anti-migratory effects by inhibiting TLR4/MyD88 signaling.

The role of TLR4/MyD88 signaling in the anti-inflammatory effects of HSR2104 was further elucidated in BV2 cells using TAK242, a specific TLR4 inhibitor. We found that TAK242 inhibited LPS-induced pro-inflammatory mediators as well as iNOS and COX2 expressions (Figure 9A–E). In addition, TAK242 abolished the LPS-induced nuclear translocation of NF-κB, cell migration, and ROS generation (Figure 9F–J). Overall, the results obtained from TAK242 treatment of LPS-stimulated BV2 cells are similar to those observed with HSR2104 treatment, confirming TLR4/MyD88 signaling is an upstream regulator of NF-κB during LPS-induced inflammatory processes. Moreover, these findings indicate that the inhibition of TLR4/MyD88 signaling by HSR2104 plays a crucial role in the inhibition of inflammatory responses, cell migration, and ROS generation in BV2 cells. Our observations suggest that HSR2104 may inhibit the interaction of TLR4 with LPS, thereby inhibiting NF-κB nuclear translocation, by which it exerts anti-inflammatory and anti-migratory effects in BV2 cells. Further studies are currently in progress to clarify this possibility.

Based on our recent reports [22,23] and the current study, the 1,2,3,4-tetrahydroquinoline derivative with an *N*-propionyl substituent (ELC-D-2), isoquinoline-1-carboxamide derivative with an *N*-hydroxyphenyl substituent (HSR1101), and HSR2104 inhibited LPS-induced pro-inflammatory mediators and cell migration in BV2 cells. Although all three compounds were commonly shown to inhibit NF-κB translocation to exert anti-inflammatory actions, their underlying mechanisms to induce the inhibition of NF-κB translocation may be different. For example, suppression of the JNK pathway was involved in the anti-inflammatory effects of ELC-D-2, whereas the inhibition of MAP kinases including ERK1/2, JNK, and p38 MAP kinase, mediated the effects of HSR1101 in BV2 cells. In the present study, we demonstrated that the suppression of ROS generation and TLR4/MyD88/NF-κB signaling pathway mediated the anti-inflammatory and anti-migratory effects of HSR2104. It would be interesting to identify all the signaling molecules of the TLR4/MyD88/NF-κB pathway mediating the effects of HSR2104 in BV2 cells. Although BV2 cells are commonly utilized as a substitute for primary microglia in many experimental settings, there have been some doubts raised as to their suitability [29,30,58]. Therefore, our findings in BV2 cells may need to be validated using primary microglia. In parallel, the in vivo study of ELC-D-2, HSR1101, and HSR2104 using various animal models such as formalin-induced paw edema and licking tests is also necessary to elucidate and compare their anti-inflammatory effects.

**Figure 10:**
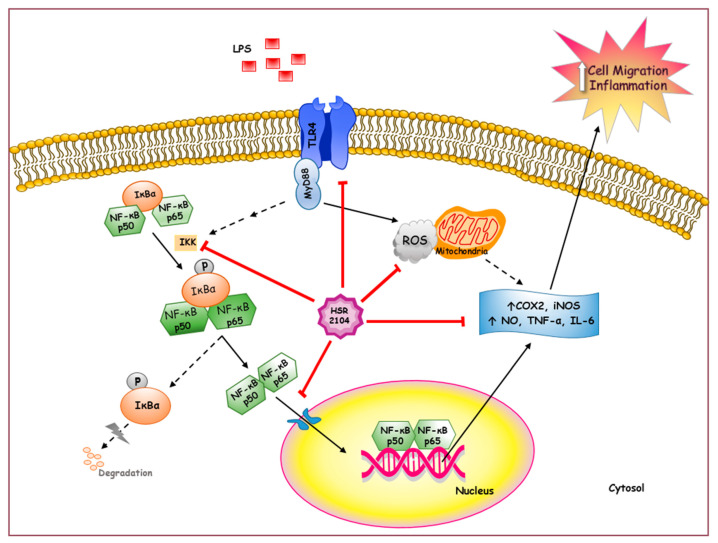
A schematic diagram illustrating the potential targets of HSR2104 to inhibit inflammatory responses and cell migration in BV2 cells. The solid lines indicate the pathways examined in this study, and the broken lines indicate the pathways not examined. The red blocked arrows indicate the pathways inhibited by HSR2104. TLR4, Toll-like receptor 4; MyD88, myeloid differentiation factor 88; ROS, reactive oxygen species; IKK, IκB kinase; IκBα, inhibitory kappa Bα; NF-κB, nuclear factor-kappa B; iNOS, inducible nitric oxide synthase; COX2, cyclooxygenase-2; IL-6, interleukin-6; TNF-α, tumor necrosis factor-α; NO, nitric oxide.

A schematic diagram illustrating the potential targets of HSR2104 to inhibit inflammatory responses and cell migration in BV2 cells is shown (Figure 10). Briefly, HSR2104 potently inhibited the LPS-induced production of pro-inflammatory mediators including NO, TNF-α, and IL-6, possibly through suppression of iNOS and COX2 expressions. In addition, HSR2104 markedly inhibited LPS-stimulated cell migration and intracellular ROS generation. Moreover, HSR2104 suppressed the LPS-induced TLR4/MyD88 expression, by which it led to subsequent inhibitions of IκBα phosphorylation and NF-κB translocation. Collectively, our data indicate that HSR2104 exhibits anti-inflammatory and anti-migratory effects in LPS-treated BV2 cells via the suppression of ROS generation and the TLR4/MyD88/NF-κB signaling pathway. Based on these findings, HSR2104 may exert beneficial effects on neuroinflammatory responses and microglial cell migration involved in the pathogenesis of various neurodegenerative disorders.

## 4. Materials and Methods

### 4.1. Synthesis of 1,8-Naphthyridine-2-Carboxamide Derivatives

A series of 1,8-naphthyridine-2-carboxamide derivatives with various substituents (HSR2101-HSR2113) was generated, according to the procedures of our previous report on the synthesis of isoquinoline-1-carboxamide derivatives [23]. In brief, the amidation reaction was accomplished by the treatment of the starting 1,8-naphthyridine-2-carboxylic acid with appropriate anilines. The ideal condition to obtain a good yield of HSR2101-2113 was the presence of a pair reagents 1,1′-carbonyldiimidazole (CDI) and anhydrous dichloromethane (DCM) at reflux for 12 h.

**Scheme 1:**
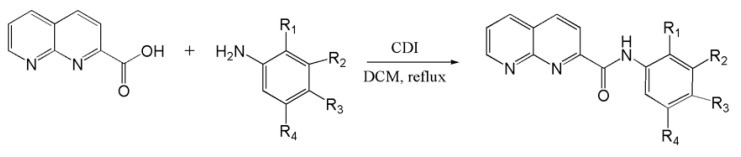
Synthesis of 1,8-naphthyridine-2-carboxamide derivatives.

The synthesis of *N*-substitutedphenyl-1,8-naphthyridine-2-carboxamide derivatives are outlined in Scheme 1. Analytical and spectral data of these compounds (HSR2101–HSR2113) are provided in the Supplementary Materials.

For our experiments, a 10 mM stock solution of each compound was prepared in dimethyl sulfoxide (DMSO), which was serially diluted to provide the indicated concentrations of each compound tested in this study.

### 4.2. Chemicals and Reagents

Dulbecco’s modified Eagle medium (DMEM), Alexa Fluor 488-conjugated anti-mouse immunoglobulin G (IgG), and 4′,6 diamidino-2-phenylindole dihydrochloride (DAPI) were obtained from Thermo Scientific (Rockford, IL, USA). Antibiotic-antimycotic reagent and fetal bovine serum (FBS) were purchased from Gibco BRL (Grand Island, NY, USA). TAK242, LPS (*Escherichia coli*, serotype 011:B4), 3-(4,5-dimethyldiazol-2-yl)-2,5-diphenyltetrazolium bromide (MTT), anti-β-actin antibody, and dimethyl sulfoxide (DMSO) were obtained from Sigma-Aldrich (St. Louis, MO, USA). Antibodies specifically recognizing TLR4, phosphorylated IκBα, and lamin B1 were supplied by Abcam (Cambridge, MA, USA), and antibodies recognizing iNOS, COX2, MyD88, NF-κB p65, non-phospho-IκBα, and horseradish peroxidase (HRP)-conjugated anti-rabbit and anti-mouse IgG were purchased from Cell Signaling Technology (Danvers, MA, USA).

### 4.3. BV2 Cell Culture, Treatment of Cells, and Determination of Cell Viability

BV2 microglial cells were cultured in DMEM supplemented with 10% FBS and 1% antibiotics at 37 °C in an incubator with 95% O_2_/5% CO_2_ as previously described [22,23]. The cells were seeded into 24-well plates at a density of 2.5 × 10^5^ cells/well and incubated at 37 °C for 24 h. To evaluate any potential cytotoxicity of test compounds in BV2 cells, the cultured cells were gently washed with phosphate-buffered saline (PBS) and treated with a series of 1,8-naphthyridine-2-carboxamide derivatives at the concentrations of 30 and 100 µM for 24 h. Control cells were treated with serum-free media containing 1% DMSO instead.

After treatment, the viability of cells was determined using an MTT assay, as described previously [59] with minor modifications. In brief, the cells were incubated with MTT solution at a final concentration of 0.5 mg/mL at 37 °C for 3 h. Then, supernatants were gently removed from each well and replaced with DMSO to dissolve blue formazan crystals. The absorbance was measured at a wavelength of 550 nm using a microplate reader (SpectraMax M2^e^, Molecular Devices, Sunnyvale, CA, USA). The cell viability was calculated as the percentage of absorbance measured in vehicle-treated control cells.

### 4.4. Measurements of NO, TNF-α, and IL-6 by Enzyme-Linked Immunosorbent Assays

Enzyme-linked immunosorbent assays (ELISA) were used to evaluate the effects of 1,8-naphthyridine-2-carboxamide derivatives on the LPS-induced production of pro-inflammatory mediators including NO, TNF-α, and IL-6. Briefly, BV2 cells were seeded into 24-well plates at a density of 2.5 × 10^5^ cells/well and incubated at 37 °C for 24 h. The cells were treated with the test compounds at the concentrations of 1, 10, 30, and 100 μM in the presence or absence of LPS (1 µg/mL). Control cells were treated with serum-free media containing 1% DMSO instead. After 24 h of treatment, the culture media were carefully collected and centrifuged at 1500 rpm at 4 °C for 10 min. The supernatants were then stored at −80 °C until use. NO concentrations in the supernatants were assessed using the Griess reagent (Promega Corporation, Madison, WI, USA) as described previously [22,23]. The levels of TNF-α and IL-6 were measured by ELISA kits (KomaBiotech, Seoul, Korea), following the manufacturer’s instructions. The absorbance was read at 450 nm on a microplate reader (SpectraMax M2^e^, Molecular Devices, Sunnyvale, CA, USA). The concentrations of NO, TNF-α, and IL-6 were calculated from the respective standard curves generated simultaneously.

### 4.5. Cell Migration Assays

#### 4.5.1. Wound Healing Assay

To assess the effect of HSR2104 on LPS-stimulated cell mobility, wound healing assays were performed as described in our previous reports [22,23] with minor modifications. Overall, BV2 cells were seeded into 24-well plates at a density of 5 × 10^5^ cells/well and incubated at 37 °C for 24 h. The cells were then wounded with a sterile scratcher and treated with HSR2104 at various concentrations in the presence or absence of LPS (1 µg/mL). Control cells were treated with serum-free media containing 1% DMSO instead. The cell migration during 24 h was determined by measuring the changes in the area of the wounds at 0 and 24 h under a Nikon phase-contrast microscope (Nikon Instruments Inc., Melville, NY, USA) [23,60], using ImageJ software version 1.49 (https://imagej.nih.gov/ij/). The degree of cell migration was expressed as a percentage of the respective vehicle-treated control cells.

#### 4.5.2. Transwell Migration Assay

To further assess the effect of HSR2104 on LPS-stimulated cell mobility, transwell migration assays were performed as described previously [22,23]. BV2 cells were seeded into the inserts (6.5 mm in diameter with an 8.0-μm pore size) of a Costar transwell system (Corning Inc., Kennebunk, ME, USA) at a density of 1 × 10^5^ cells/well and incubated at 37 °C for 24 h in DMEM supplemented with 10% FBS and 1% antibiotics. After incubation, the bottom wells were filled with HSR2104 at various concentrations with or without LPS (1 μg/mL) in serum-free media. Control cells were treated with serum-free media containing 1% DMSO instead. After 24 h of treatment, non-migrated cells were gently removed with a cotton swab and the migrated cells were fixed with 4% paraformaldehyde for 20 min, followed by permeabilization with methanol for 10 min prior to staining with 0.5% crystal violet for 10 min, as described previously [23,60]. Four random fields from each well were captured and the numbers of cells migrated through the membrane were counted under a Nikon phase-contrast microscope (Nikon Instruments Inc., Melville, NY, USA). The cell migration was expressed as a percentage of the respective vehicle-treated control cells.

### 4.6. Western Blotting

BV2 cells were plated on 35 mm dishes at a density of 1 × 10^6^ cells/dish and incubated at 37 °C for 24 h. The cells were washed with PBS and treated with a series of concentrations of HSR2104 in the presence or absence of LPS (1 µg/mL). Control cells were treated with serum-free media containing 1% DMSO instead. After treatment, the cells were washed with cold PBS and lysed in lysis buffer (10 mM Tris–HCl (pH 7.5), 150 mM NaCl, 2 mM EDTA, 4.5 mM sodium pyrophosphate, 10 mM β-glycerophosphate, 1 mM NaF, 1 mM Na_3_VO_4_, 1% (*v*/*v*) Triton X-100, 0.5% IGE-PAL CA-630 and one tablet of protease inhibitor cocktail (Roche Diagnostic GmbH, Mannheim, Germany) per 10 mL of lysis buffer) on ice for 30 min. The cell lysates were centrifuged at 14,000 rpm for 30 min at 4 °C and then the supernatants were separated and stored at −80 °C until use. To evaluate the effect of HSR2104 on the nuclear translocation of NF-κB, cytosolic and nuclear fractions were prepared using NE-PER Nuclear and Cytoplasmic Extraction Reagents (Rockford, IL, USA), following the manufacturer’s instructions. The protein concentrations were determined using a BioRad DC protein assay kit (BioRad, Hercules, CA, USA).

Western blotting analyses were performed as reported previously [22,59]. Briefly, cell lysates containing equal amounts of protein were resolved by SDS-PAGE and transferred to nitrocellulose membranes (Merck Millipore Ltd., Billerica, MA, USA) at 100 V for 90 min. The transferred membranes were blocked with 5% non-fat dried skim milk (BD Biosciences, San Jose, CA, USA) at room temperature for 90 min and incubated with primary antibodies in bovine serum albumin at 4 °C overnight. After rinsing with Tris-buffered saline containing 0.1% Tween 20, the membranes were incubated with HRP-conjugated secondary antibodies at room temperature for 120 min. Finally, blots were visualized with a Bio-Rad ChemiDoc XRS imaging system using enhanced chemiluminescence reagents (Bio-Rad, Hercules, CA, USA).

### 4.7. Immunocytochemistry

To further assess the effect of HSR2104 on LPS-induced NF-κB translocation, immunocytochemistry was performed as described previously [61] with some modifications. In brief, BV2 cells were plated on coverslips placed on the wells of 24-well plates at a density of 2.5 × 10^4^ cells/well and incubated at 37 °C for 24 h. The cells were then treated with 100 µM HSR2104 in the presence or absence of LPS (1 µg/mL) for 24 h. Control cells were treated with serum-free media containing 1% DMSO instead. After treatment, the cells were fixed with 4% paraformaldehyde for 15 min and permeabilized with 1% Triton X-100 for 5 min, followed by incubation with blocking solution of 5% goat serum for 30 min. Between each step, the cells were carefully washed three times with PBS. The cells were then incubated with anti-NF-κB p65 antibody with 1:100 dilution in blocking solution at 4 °C overnight. After washing three times with PBS, the cells were incubated with Alexa Fluor 488-conjugated secondary antibody (1:400 dilution) for 1 h at room temperature in the dark. Then, after washing three times with PBS, coverslips were removed from 24-well plates and mounted onto microscope slides (Paul Marienfeld GmbH, Lauda-Königshofen, Germany) with ProLong Gold Antifade Reagent with DAPI (Thermo Fisher Scientific Inc., Waltham, MA, USA). Fluorescent signals were visualized using a Nikon confocal laser-scanning microscope (Nikon Instruments Inc., Melville, NY, USA).

### 4.8. Measurements of Reactive Oxygen Species

The effect of HSR2104 on LPS-induced intracellular ROS generation was assessed using a DCFH-DA assay, as described previously [59,61] with some modifications. Briefly, BV2 cells were seeded into 24-well plates at a density of 1 × 10^5^ cells/well and incubated at 37 °C for 24 h. The cells were then treated with various concentrations of HSR2104 with or without LPS (1 µg/mL) for 24 h. Following treatment, the culture media were replaced with serum free media containing DCFH-DA at a final concentration of 10 µM and incubated at 37 °C for 1 h. The levels of intracellular ROS were quantitated by the fluorescence detection of dichlorofluorescein as an oxidized product of DCFH on a microplate reader (SpectraMax M2^e^, Molecular Devices, Sunnyvale, CA, USA) with an excitation wavelength of 490 nm and emission wavelength of 520 nm. The levels of ROS production were expressed as percentages of the vehicle-treated control cells. The fluorescent signals were visualized using a fluorescence microscope (Nikon Instruments Inc., Melville, NY, USA).

### 4.9. Statistical Analyses

All experiments were performed at least three times and quantitative data were expressed as the means ± SEM. Statistical significance was analyzed by one-way analysis of variance (ANOVA) using Sigma Plot 12.5 software (Systat Software Inc., San Jose, CA, USA). A *p* value < 0.05 was considered statistically significant. IC_50_ values were calculated by non-linear regression using GraphPad Prism 5 (GraphPad Software Inc., La Jolla, CA, USA).

## 5. Conclusions

In conclusion, thirteen novel compounds of 1,8-naphthyridine-2-carboxamide derivatives with *N*-hydroxyphenyl, *N*-methoxyphenyl, *N*-(trifluoromethyl)phenyl, *N*-bis(trifluoromethyl)phenyl, or *N*-chlorophenyl substituents were synthesized and evaluated for anti-inflammatory and anti-migratory effects using LPS-treated BV2 microglia cells. Among the compounds tested, HSR2104 with an *N*-methoxyphenyl substituent at the R1 position, exhibited the most potent inhibitory effects on the production of pro-inflammatory mediators including NO, TNF-α, and IL-6. HSR2104 inhibited the expressions of iNOS and COX2 as well as cell migration in LPS-treated BV2 cells. In addition, it markedly suppressed LPS-induced intracellular ROS production. Moreover, HSR2104 abated the LPS-triggered nuclear translocation of NF-κB through the inhibition of IκBα phosphorylation. It was further demonstrated that HSR2104 inhibited LPS-induced TLR4/MyD88/NF-κB signaling. Collectively, our data indicate that HSR2104 exhibits anti-inflammatory and anti-migratory effects in LPS-treated BV2 cells via the suppression of ROS production and the TLR4/MyD88/NF-κB signaling pathway. Based on these findings, HSR2104 may exert beneficial inhibitory effects on neuroinflammatory responses and microglial cell migration associated with the pathogenesis of neurodegenerative disorders including AD and PD.

## Figures and Tables

**Table 1:** IC_50_ values of the selected 1,8-naphthyridine-2-carboxamide derivatives for the inhibition of NO, TNF-α, and IL-6 production in LPS-treated BV2 cells.

Inflammatory Mediators	IC_50_ Values (µM) *							
	HSR2101	HSR2102	HSR2103	HSR2104	HSR2105	HSR2106	HSR2107	HSR2113
NO	>100	32.13	45.38	25.43	50.72	57.02	28.03	26.49
TNF-α	30.47	52.09	59.36	33.24	44.61	57.21	37.26	91.93
IL-6	>100	55.74	78.91	32.03	50.50	29.16	96.06	27.98

* IC_50_ value represents the concentration of each compound required to inhibit LPS-stimulated NO, TNF-α, and IL-6 production by 50%.

## Supplementary Materials

Supplementary Materials can be found at https://www.mdpi.com/1422-0067/22/5/2527/s1.

## Abbreviations

COX2	Cyclooxygenase-2
IκBs	Inhibitors of kappa B
IL	Interleukin
iNOS	Inducible nitric oxide synthase
LPS	Lipopolysaccharide
MyD88	Myeloid differentiation factor 88
NF-κB	Nuclear factor-kappa B
NO	Nitric oxide
ROS	Reactive oxygen species
TLR4	Toll-like receptor 4
TNF-α	Tumor necrosis factor-alpha
