# Supplementary material for: A Novel 1,8-Naphthyridine-2-Carboxamide Derivative Attenuates Inflammatory Responses and Cell Migration in LPS-Treated BV2 Cells via the Suppression of ROS Generation and TLR4/Myd88/NF-κB Signaling Pathway
Source: Int J Mol Sci. 2021 Mar 3;22(5):2527. doi: 10.3390/ijms22052527 (PMC7959294; doi:10.3390/ijms22052527)

Supplementary Information

General Information

All the reactions were carried out in oven dried glassware with freshly distilled dry solvents under anhydrous conditions unless otherwise indicated and all commercial chemicals were used as obtained. Evaporation of organic solutions was achieved by rotary evaporation with a water bath temperature below 40°C. All the products obtained were purified by column chromatography using silica gel (100-200 mesh). Thin layer chromatography was performed on E Merck silica gel GF-254 pre-coated plates; identification was performed under UV illumination. ^{1}H and ^{13}C NMR were recorded in JEOL 400 and 101 MHz spectrometer respectively. The chemical shifts are reported in ppm downfield to TMS (δ = 0) for ^{1}H NMR and relative to the central $CDCl_3$ resonance (δ = 77.0) for ^{13}C NMR. Data are reported as follows: chemical shift in ppm (d), multiplicity (s = singlet, d = doublet, t = triplet, m = multiplet), coupling constant (Hz), and integration. High resolution mass spectra (HRMS) recorded on LCQ Fleet-Thermo Scientifics.

General Synthetic Procedures

General procedure for the synthesis of *N*-(substitutedphenyl)-1,8-naphthyridine-2-carboxamide derivatives (HSR2101-2113)

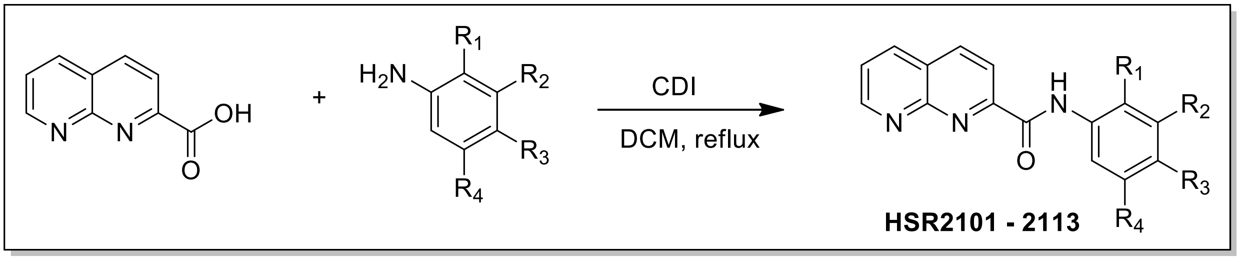

Under a nitrogen atmosphere, a mixture of 1,8-naphthyridine-2-carboxylic acid (600 mg, 3.45 mmol) and 1,1'-carbodiimidazole (1.68 g, 10.34 mmol) in anhydrous DCM (20 mL) was stirred at heated at reflux for 2 h. After adding 2-aminophenol (2.12 g, 17.22 mmol), the reaction solution was further stirred at reflux for 12 h. The crude mixture was diluted with water, and then extracted with ethyl acetate (3 x 20 mL) and chloromethane (3 x 20 mL), respectively. The combined organic layer was dried over anhydrous $MgSO_4$ and the solvent was evaporated. The residue was purified by flash column chromatography on silica gel (EtOAc/hexane = 1:1) to afford the corresponding *N*-substitutedphenyl-1,8-naphthyridine-2-carboxamides (**HSR2101** – **HSR2113**).

Analytical Data

_N_-(2-Hydroxyphenyl)-1,8-naphthyridine-2-carboxamide (HSR2101)

Brown solid; 25% yield; ^{1}H NMR (400 MHz, DMSO-d_6) δ 10.67 (s, 1H), 10.52 (s,1H), 9.23 (q, 1H, J = 2.3 Hz), 8.78 (d, 1H, J = 8.2 Hz), 8.63 (d, 1H, J = 8.2 Hz), 8.44-8.40 (m, 2H), 7.79 (m, 1H), 7.02-6.98 (m, 2H), 6.88 (m, 1H); ^{13}C NMR (100 MHz, DMSO-d_6) δ 161.4, 155.7, 154.2, 152.7, 147.3, 141.0, 138.3, 126.6, 124.9, 124.8, 119.8, 119.7, 115.3; HRMS m/z $[M+H]^+$ calculated for $C_{15}H_{12}N_3O_2$: 266.0924; found: 266.0914.

_N_-(3-Hydroxyphenyl)-1,8-naphthyridine-2-carboxamide (HSR2102)

Yellow solid; 19% yield; ^{1}H NMR (400 MHz, DMSO-d_6) δ 10.61 (s, 1H), 9.45 (s, 1H), 9.23 (d, 1H, J = 1.8 Hz), 8.73 (d, 1H, J = 8.2 Hz), 8.61 (d, 1H, J = 8.2 Hz), 8.31 (d, 1H, J = 8.2 Hz), 7.77

(m, 1H), 7.52 (t, 1H, *J* = 2.3 Hz), 7.30 (m, 1H), 7.17 (t, 1H, *J* = 7.8 Hz), 6.57 (d, 1H, *J* = 7.8 Hz); ^{13}C NMR (100 MHz, DMSO-d_6) δ 162.9, 158.2, 155.5, 154.3, 153.5, 140.6, 139.7, 138.3, 130.0, 124.5, 124.3, 120.3, 111.9, 111.6, 107.9; HRMS m/z $[M+H]^+$ calculated for $C_{15}H_{12}N_3O_2$: 266.0924; found: 266.0916.

***N*-(4-Hydroxyphenyl)-1,8-naphthyridine-2-carboxamide (HSR2103)**

Yellow solid; 35% yield; ^{1}H NMR (400 MHz, DMSO-d_6) δ 10.57 (s, 1H), 9.35 (s, 1H), 9.23 (m, 1H), 8.73 (d, 1H, *J* = 8.2 Hz), 8.61 (d, 1H, *J* = 8.2 Hz), 8.33 (d, 1H, *J* = 8.2 Hz), 7.78-7.72 (m, 3H), 6.80 (d, 2H, *J* = 9.2 Hz); ^{13}C NMR (100 MHz, DMSO-d_6) δ 162.4, 155.4, 154.6, 154.4, 153.7, 140.5, 138.3, 130.4, 124.4, 124.2, 122.6, 120.3, 115.7; HRMS m/z $[M+H]^+$ calculated for $C_{15}H_{12}N_3O_2$: 266.0924; found: 266.0917.

***N*-(2-Methoxyphenyl)-1,8-naphthyridine-2-carboxamide (HSR2104)**

Yellow solid; 68% yield; ^{1}H NMR (400 MHz, $CDCl_3$) δ 10.73 (s, 1H), 9.27 (m, 1H), 8.61 (d, 1H, *J* = 8.2 Hz), 8.57 (d, 1H, *J* = 8.2 Hz), 8.45 (d, 1H, *J* = 8.0 Hz), 8.35 (d, 1H, *J* = 7.8 Hz), 7.64 (m, 1H), 7.14 (m. 1H), 7.04 (m, 1H), 6.97 (d, 1H, *J* = 7.8 Hz), 4.00 (s, 3H); ^{13}C NMR (100 MHz, $CDCl_3$) δ 161.6, 154.5, 153.7, 149.4, 139.2, 137.7, 127.1, 124.6, 124.4, 120.9, 120.5, 120.4, 110.2, 58.9; HRMS m/z $[M+H]^+$ calculated for $C_{16}H_{14}N_3O_2$: 280.1081; found: 280.1075.

***N*-(3-Methoxyphenyl)-1,8-naphthyridine-2-carboxamide (HSR2105)**

Yellow solid; 75% yield; ^{1}H NMR (400 MHz, $CDCl_3$) δ 10.27 (s, 1H), 9.25 (m, 1H), 8.56 (d, 1H, *J* = 8.2 Hz), 8.46 (d, 1H, *J* = 8.7 Hz), 8.35 (d, 1H, *J* = 8.2 Hz), 7.65 (m, 1H), 7.58 (t, 1H, *J* = 2.3 Hz), 7.41 (m, 1H), 7.32 (t, 1H, *J* = 8.2 Hz), 6.75 (m, 1H), 3.87 (s, 3H); ^{13}C NMR (100 MHz,

$CDCl_3$) δ 161.5, 160.3, 154.8, 154.2, 152.9, 139.4, 138.8, 137.4, 129.9, 124.4, 123.5, 120.2, 112.2, 110.0, 105.3, 55.4; HRMS m/z $[M+H]^+$ calculated for $C_{16}H_{14}N_3O_2$: 280.1081; found: 280.1075.

***N*-(4-Methoxyphenyl)-1,8-naphthyridine-2-carboxamide (HSR2106)**

Yellow solid; 73% yield; ^1H NMR (400 MHz, $CDCl_3$) δ 10.17 (s, 1H), 9.23 (m, 1H), 8.55 (d, 1H, *J* = 8.7 Hz), 8.44 (d, 1H, *J* = 8.7 Hz), 8.33 (d, 1H, *J* = 8.2 Hz), 7.80-7.76 (m, 2H), 7.63 (m, 1H), 6.95 (m, 1H), 3.83 (s, 3H); ^{13}C NMR (100 MHz, $CDCl_3$) δ 161.2, 156.7, 154.2, 153.2, 139.3, 137.5, 130.9, 124.4, 123.4, 121.6, 120.3, 114.4, 55.6; HRMS m/z $[M+H]^+$ calculated for $C_{16}H_{14}N_3O_2$: 280.1081; found: 280.1075.

***N*-(2-(Trifluoromethyl)phenyl)-1,8-naphthyridine-2-carboxamide (HSR2107)**

White solid; 87% yield; ^1H NMR (400 MHz, $CDCl_3$) δ 10.76 (s, 1H), 9.30 (m, 1H), 8.54 (d, 1H, *J* = 8.2 Hz), 8.48-8.45 (m, 2H), 8.35 (d, 1H, *J* = 8.2 Hz), 7.72 (d, 1H, *J* = 8.0 Hz), 7.67-7.63 (m, 2H), 7.32 (t, 1H, *J* = 7.3 Hz); ^{13}C NMR (100 MHz, $CDCl_3$) δ 162.3, 155.2, 154.2, 152.3, 139.5, 137,3, 133.0, 126.4, 124.9, 124.7, 124.2, 123.6, 120.1; HRMS m/z $[M+H]^+$ calculated for $C_{16}H_{11}F_3N_3O$: 318.0849; found: 318.0847.

***N*-(3-(Trifluoromethyl)phenyl)-1,8-naphthyridine-2-carboxamide (HSR2108)**

White solid; 85% yield; ^1H NMR (400 MHz, $CDCl_3$) δ 10.41 (s, 1H), 9.26 (m, 1H), 8.57 (d, 1H, *J* = 8.2 Hz), 8.48 (d, 1H, *J* = 8.2 Hz), 8.36 (d, 1H, *J* = 8.2 Hz), 8.27 (s, 1H), 8.00 (d, 1H, *J* = 7.8 Hz), 7.66 (m, 1H), 7.55 (t, 1H, *J* = 7.8 Hz), 7.44 (d, 1H, *J* = 7.8 Hz); ^{13}C NMR (100 MHz, $CDCl_3$) δ 161.8, 155.0, 154.2, 152.4, 139.6, 138.2, 137.5, 129.6, 123.7, 122.9, 121.3, 120.2,

116.9; HRMS m/z $[M+H]^+$ calculated for $C_{16}H_{11}F_3N_3O$: 318.0849; found: 318.0845.

***N*-(4-(Trifluoromethyl)phenyl)-1,8-naphthyridine-2-carboxamide (HSR2109)**

White solid; 81% yield; ^1H NMR (400 MHz, $CDCl_3$) δ 10.43 (s, 1H), 9.27 (m, 1H), 8.56 (d, 1H, *J* = 8.2 Hz), 8.40 (d, 1H, *J* = 8.7 Hz), 8.37 (d. 1H, *J* = 8.2 Hz), 8.00 (d, 2H, *J* = 8.2 Hz), 7.69-7.65 (m, 3H); ^{13}C NMR (100 MHz, $CDCl_3$) δ 161.9, 155.0, 154.1, 152.4, 140.7, 137.5, 134.6, 126.5, 126.2, 124.6, 123.7, 120.2, 119.7; HRMS m/z $[M+H]^+$ calculated for $C_{16}H_{11}F_3N_3O$: 318.0849; found: 318.0843.

***N*-(3,5-Bis(trifluoromethyl)phenyl)-1,8-naphthyridine-2-carboxamide (HSR2110)**

White solid; 88% yield; ^1H NMR (400 MHz, $CDCl_3$) δ 10.60 (s, 1H), 9.28 (m, 1H), 8.55 (q, 2H, *J* = 8.2 Hz), 8.40-8.37 (m, 3H), 7.70-7.67 (m, 2H); ^{13}C NMR (100 MHz, $CDCl_3$) δ 162.0, 155.1, 154.0, 151.8, 139.9, 139.1, 137.6, 132.3, 132.5, 124,8, 123.9, 120.2, 119.7, 117.9; HRMS m/z $[M+H]^+$ calculated for $C_{17}H_9F_6N_3O$: 386.0723; found: 386.0743.

***N*-(2-Chlorophenyl)-1,8-naphthyridine-2-carboxamide (HSR2111)**

White solid; 86% yield; ^1H NMR (400 MHz, $CDCl_3$) δ 10.87 (s, 1H), 9.27 (m, 1H), 8.62 (d, 1H, *J* = 8.2 Hz), 8.53 (d, 1H, *J* = 8.7 Hz), 8.45 (d, 1H, *J* = 8.7 Hz), 8.32 (d, 1H, *J* = 8.2 Hz), 7.63 (m, 1H), 7.46 (d, 1H, *J* = 7.8 Hz), 7.36 (t, 1H, *J* = 8.7 Hz), 7.13 (t, 1H, *J* = 7.8 Hz); ^{13}C NMR (100 MHz, $CDCl_3$) δ 161.8, 155.1, 152.7, 139.5, 137.4, 134.6, 129.5, 127.7, 125.2, 124,6, 124.4, 123.6, 121.7, 120.2; HRMS m/z $[M+H]^+$ calculated for $C_{15}H_{11}ClN_3O$:284.0585; found: 284.0582.

***N*-(3-Chlorophenyl)-1,8-naphthyridine-2-carboxamide (HSR2112)**

White solid; 85% yield; ^{1}H NMR (400 MHz, $CDCl_3$) δ 10.30 (s, 1H), 9.24 (d, 1H, J = 2.3 Hz), 8.53 (d, 1H, J = 1.8 Hz), 8.46 (d, 1H, J = 8.7 Hz), 8.34 (d, 1H, J = 8.2 Hz), 8.05 (t. 1H, J = 1.8 Hz), 7.66-7.62 (m, 2H), 7.33 (t, 1H, J = 8.23), 7.15 (m, 1H); ^{13}C NMR (100 MHz, $CDCl_3$) δ 161.6, 154.7, 153.9, 152.6, 139.5, 138.8, 137.7, 134.9, 130.2, 124.8, 124.6, 123.7, 120.4, 120.2, 118.0; HRMS m/z $[M+H]^+$ calculated for $C_{15}H_{11}ClN_3O$:284.0585; found: 284.0585.

***N*-(4-Chlorophenyl)-1,8-naphthyridine-2-carboxamide (HSR2113)**

White solid; 80% yield; ^{1}H NMR (400 MHz, $CDCl_3$) δ 10.29 (s, 1H), 9.25 (q, 1H, J = 1.8 Hz), 8.55 (d, 1H, J = 8.2 Hz), 8.47 (d, 1H, J = 8.7 Hz), 8.35 (d, 1H, J = 8.2 Hz), 7.85-7.81 (m, 2H), 7.65 (d, 1H, J = 8.2 Hz), 7.40-7.36 (m, 2H); ^{13}C NMR (100 MHz, $CDCl_3$) δ 161.6, 155.0, 154.2, 152.7, 139.5, 137.4, 136.3, 129.7, 129.3, 124.5, 123.6, 121.6, 120.2; HRMS m/z $[M+H]^+$ calculated for $C_{15}H_{11}ClN_3O$:284.0585; found: 284.0583.

<u>Spectra of all synthesized compounds</u>

^{1}H and ^{13}C NMR Spectra of HSR2101

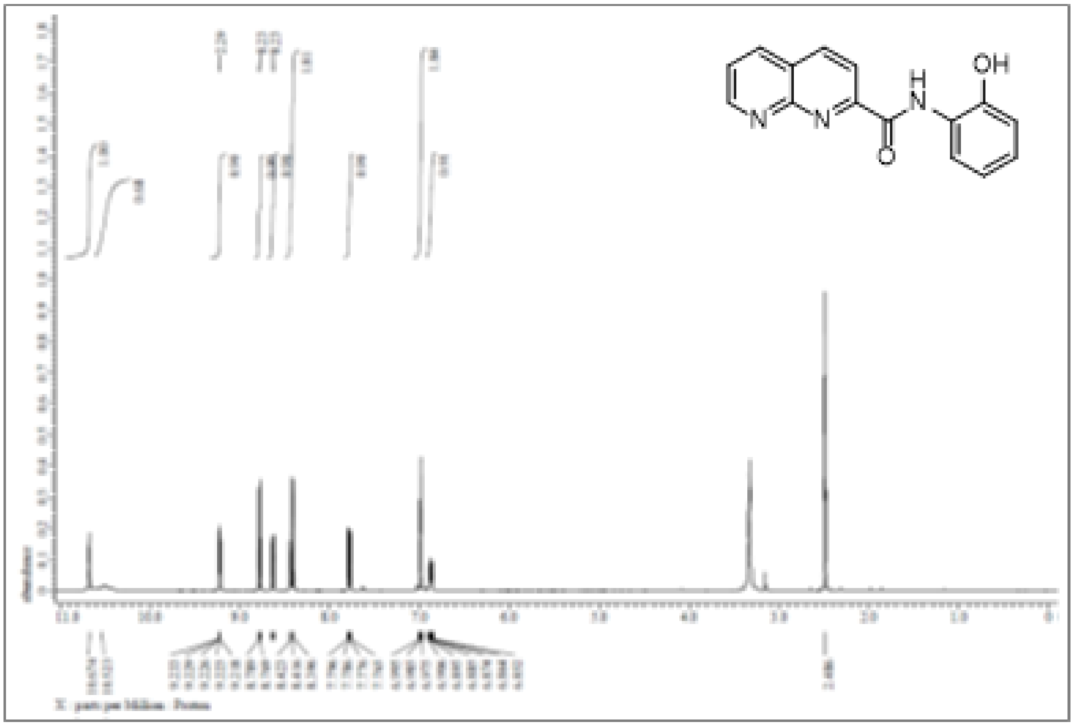

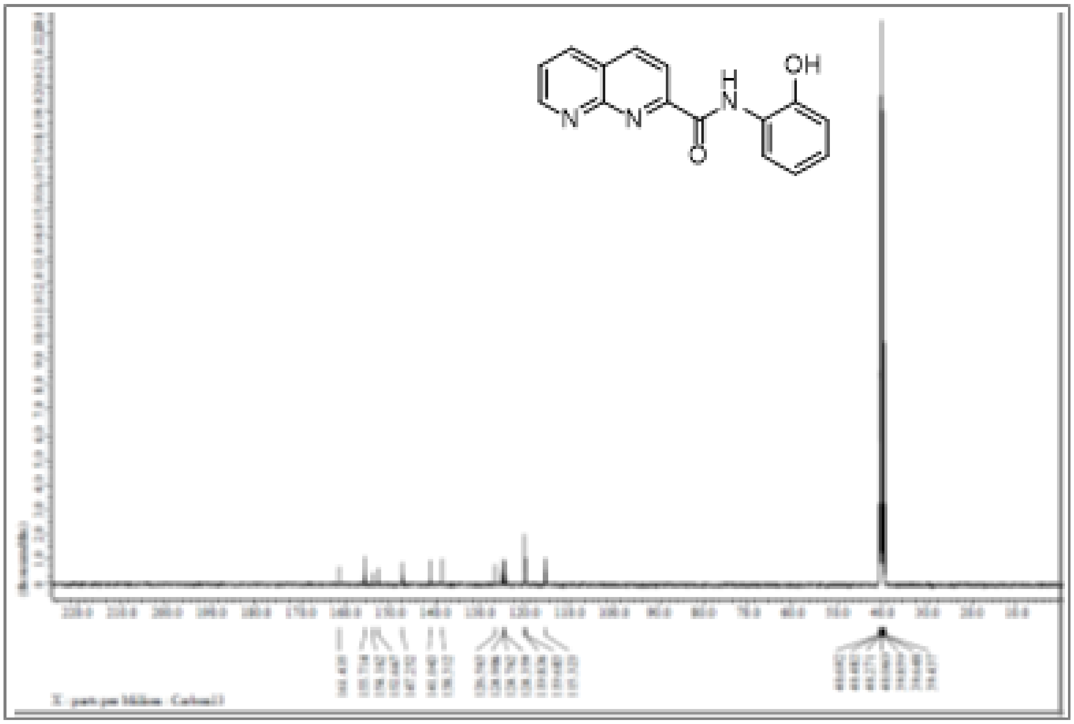

¹H and ¹³C NMR Spectra of HSR2102

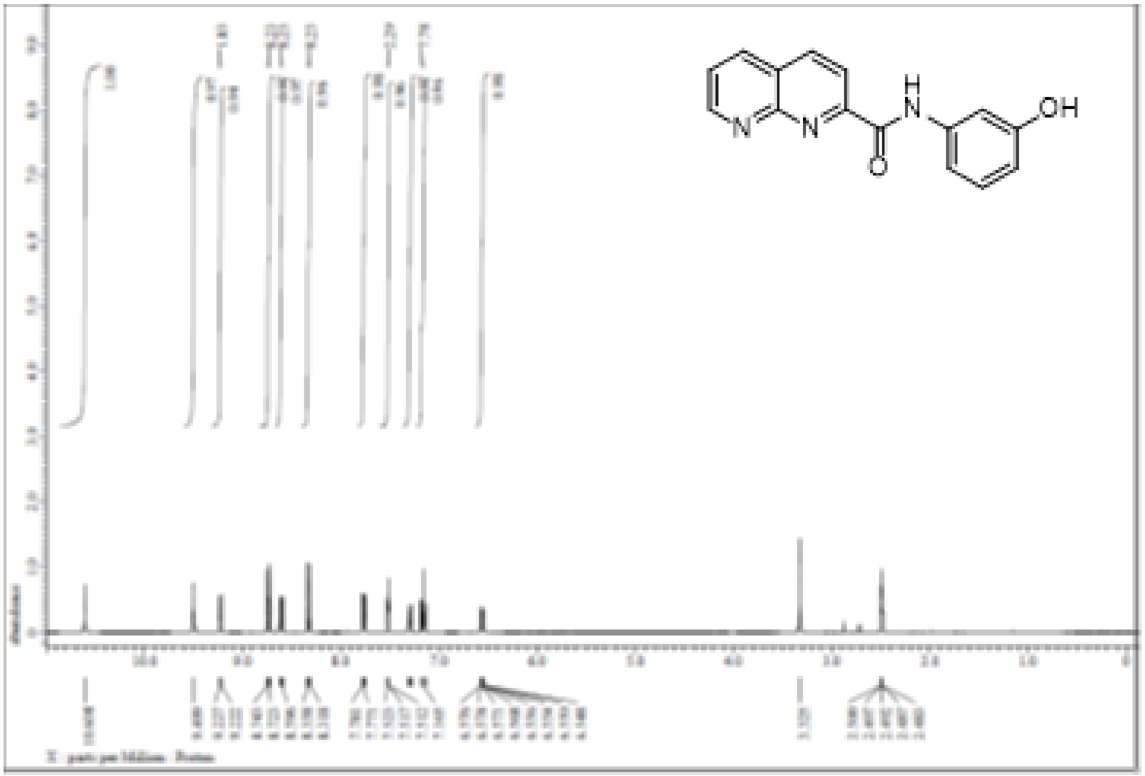

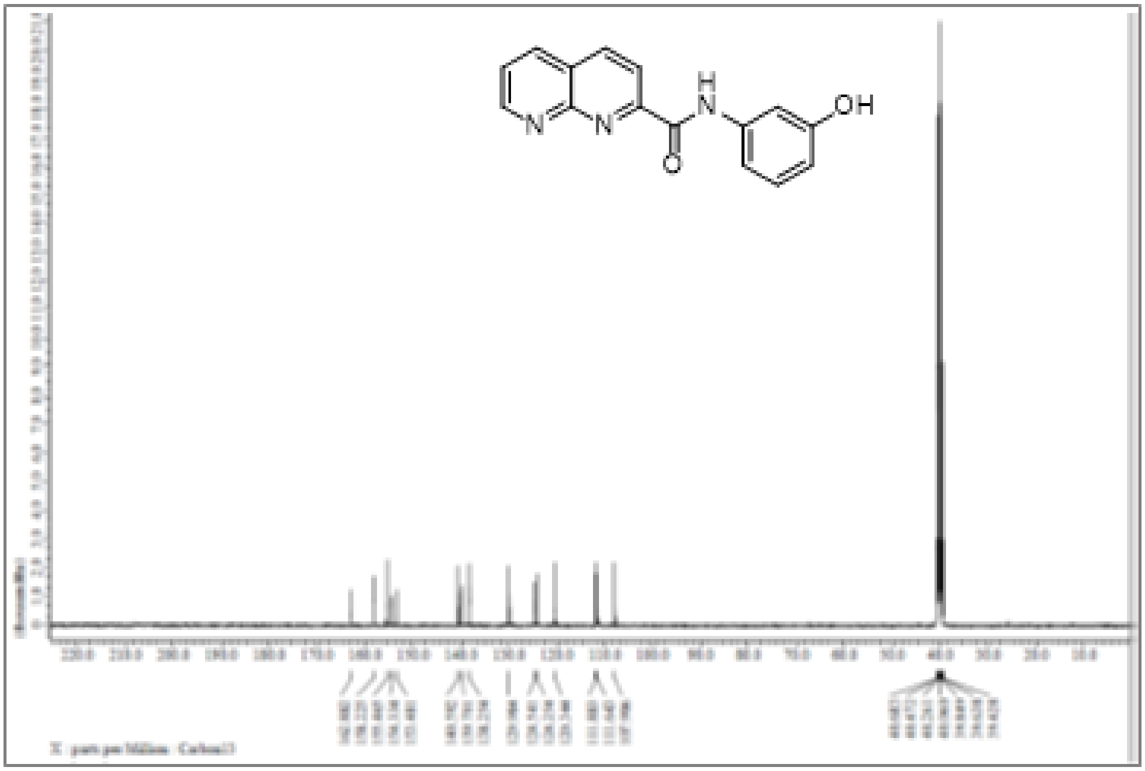

^{1}H and ^{13}C NMR Spectra of HSR2103

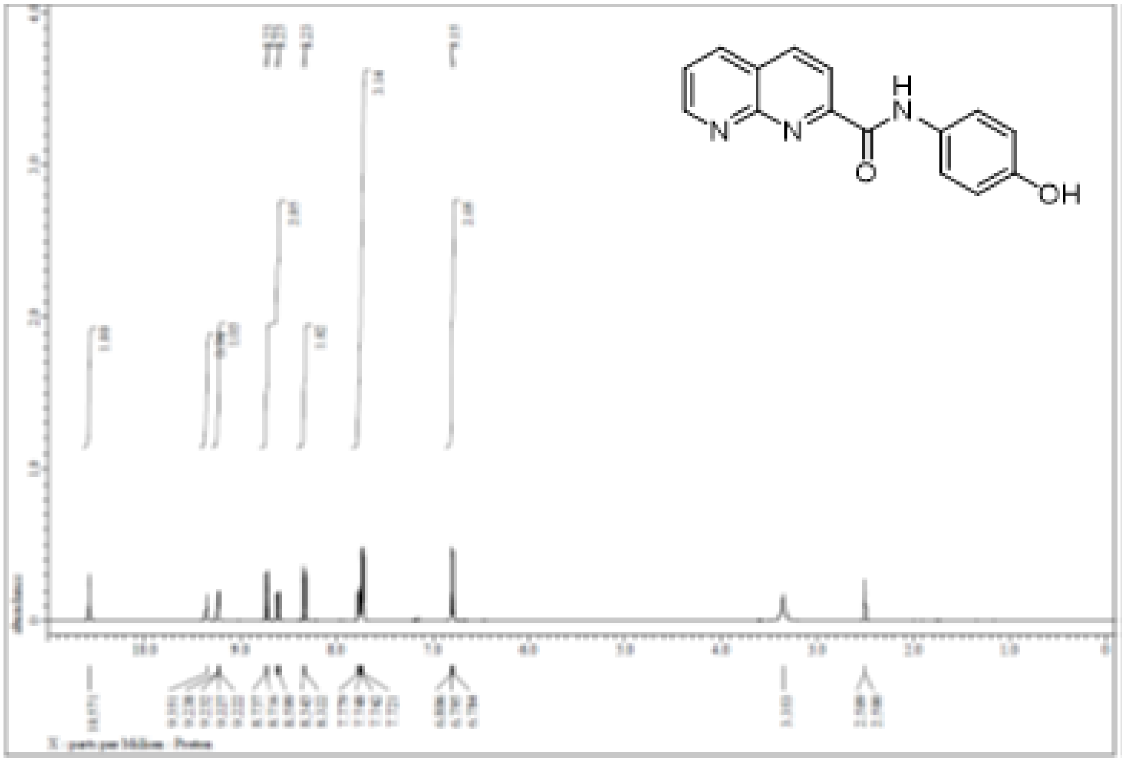

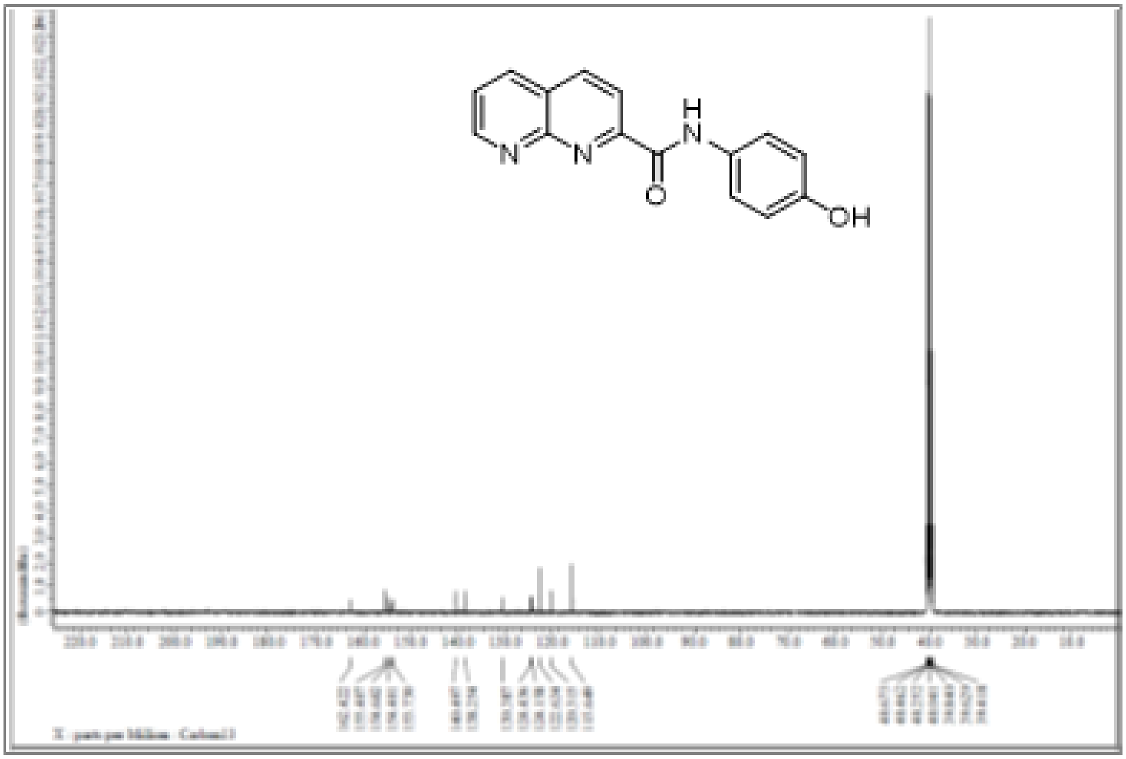

^{1}H and ^{13}C NMR Spectra of HSR2104

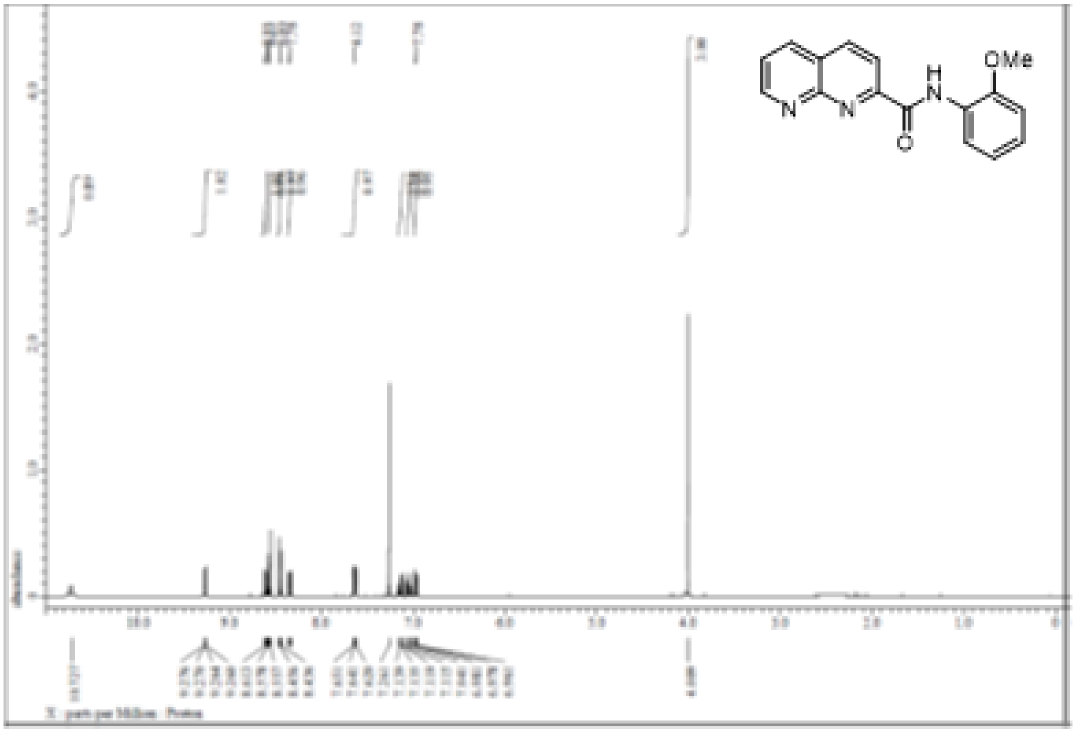

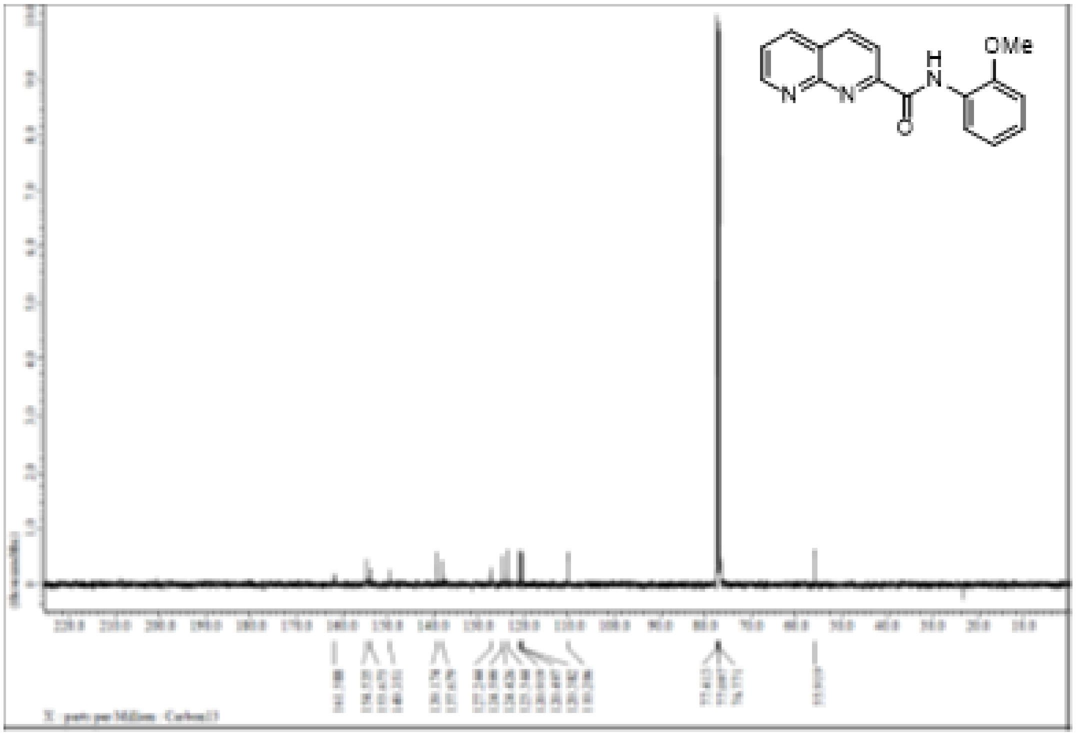

^{1}H and ^{13}C NMR Spectra of HSR2105

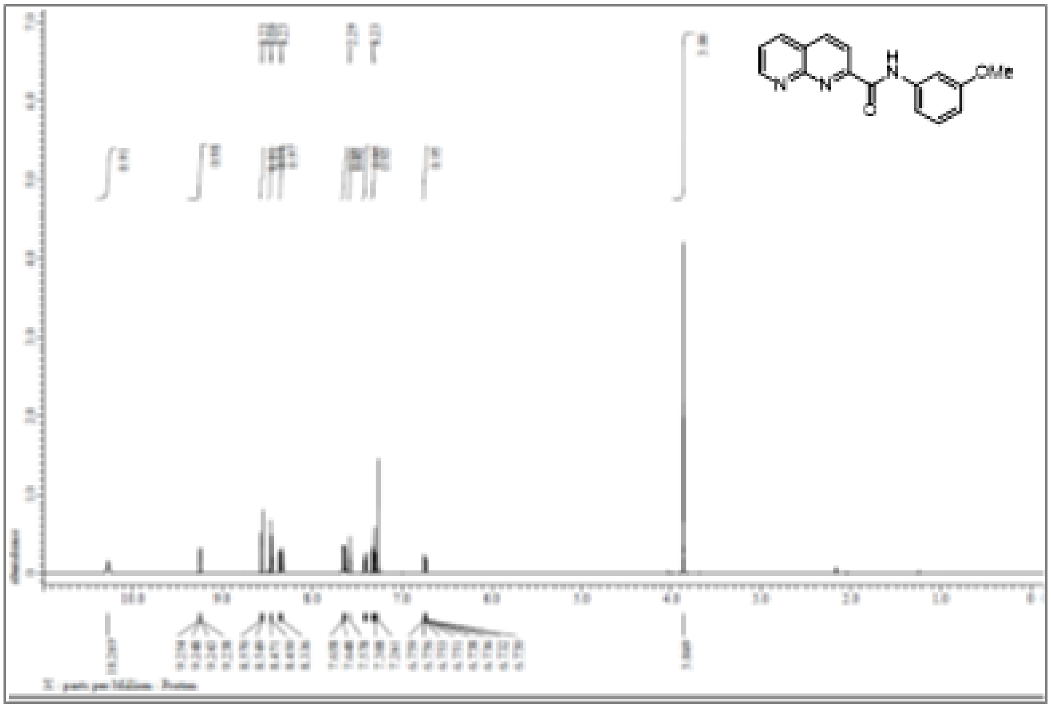

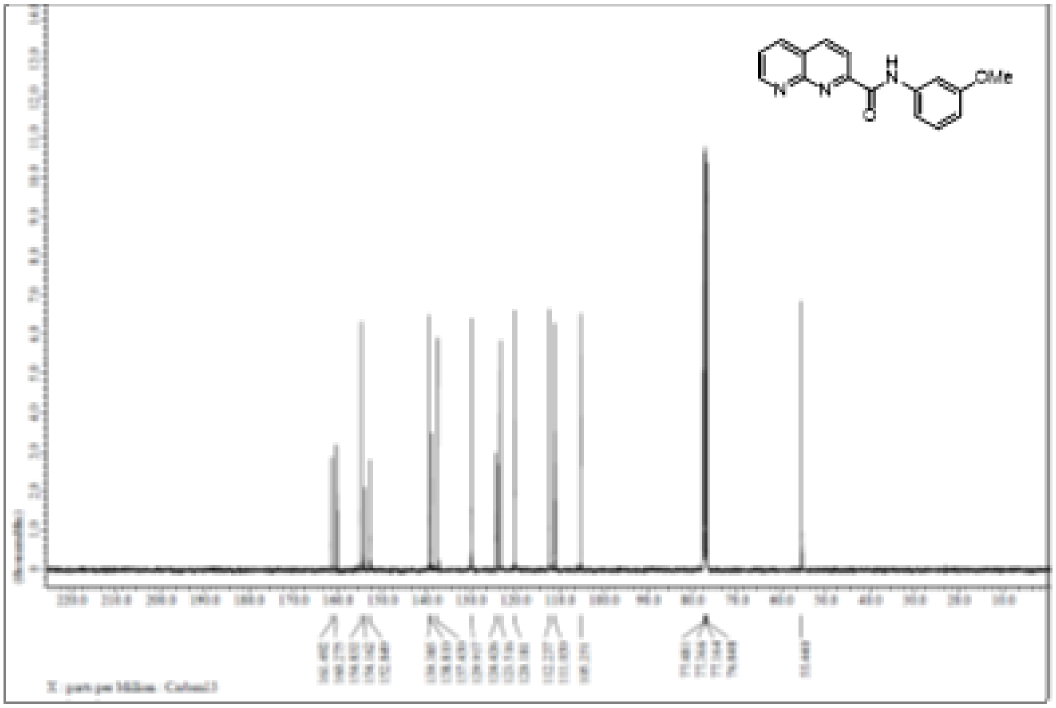

^{1}H and ^{13}C NMR Spectra of HSR2106

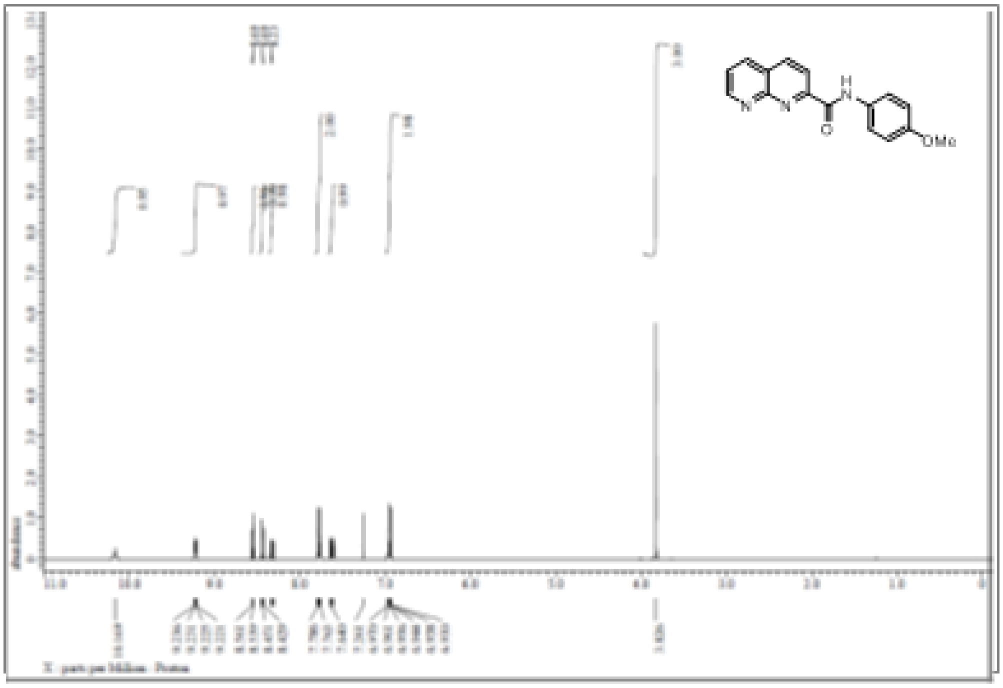

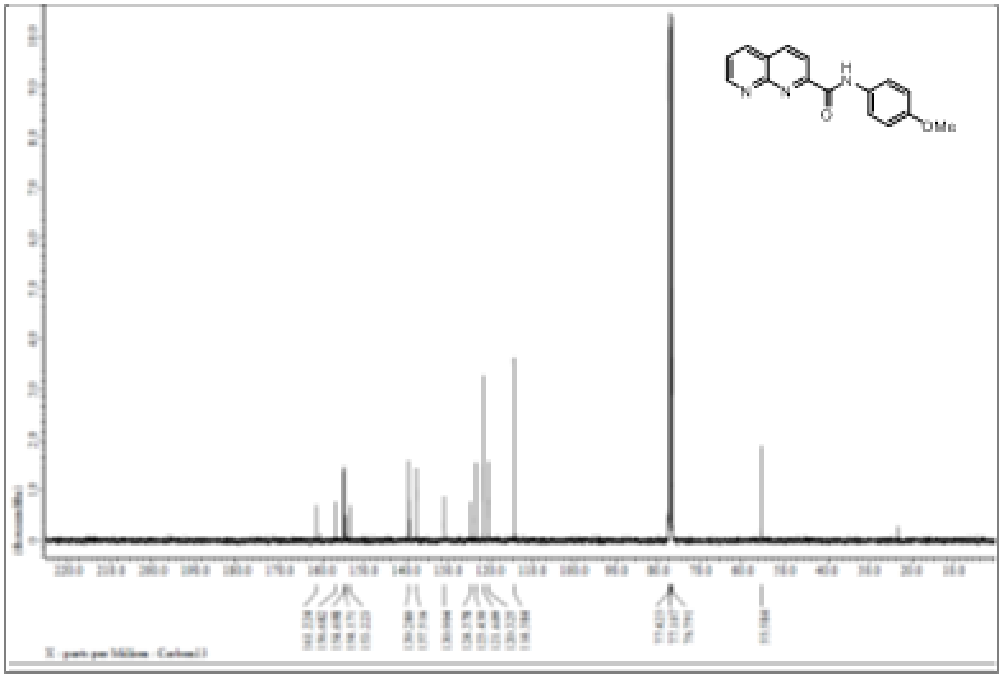

^{1}H and ^{13}C NMR Spectra of HSR2107

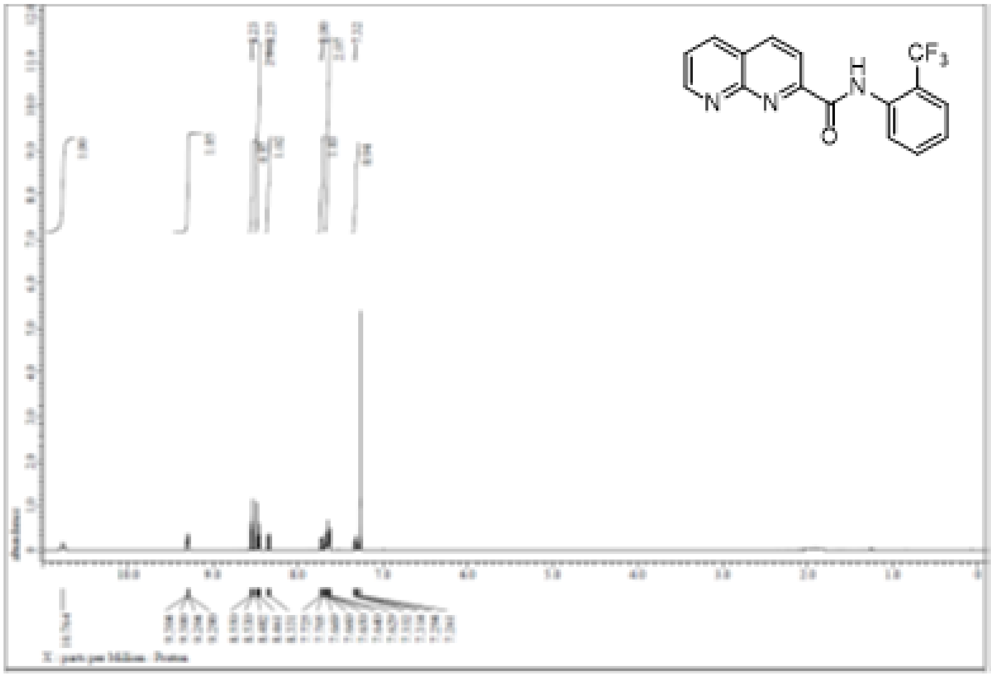

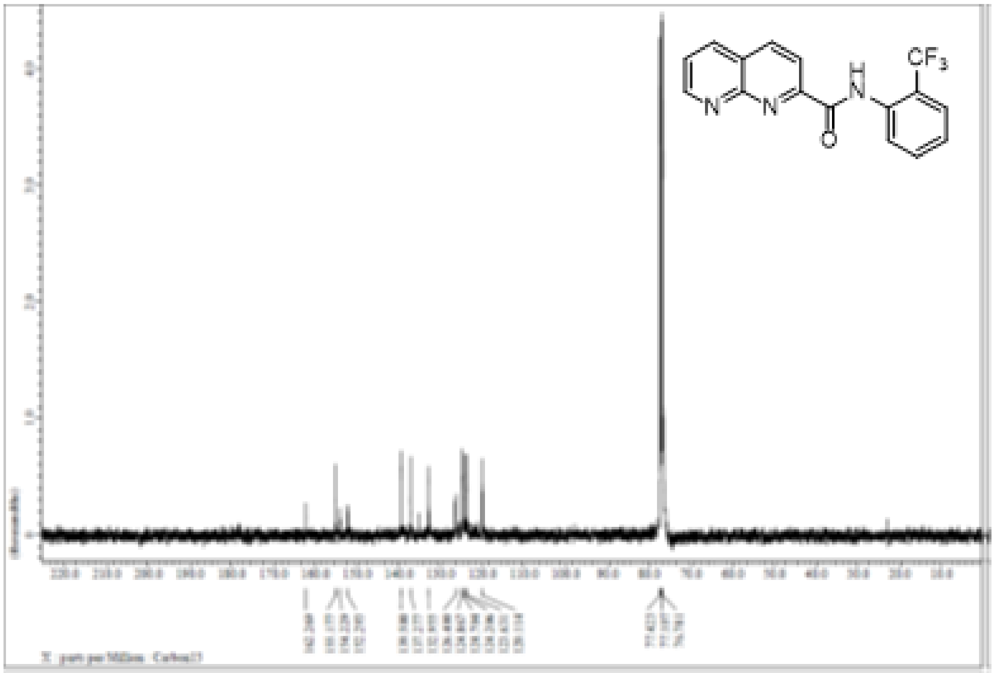

^{1}H and ^{13}C NMR Spectra of HSR2108

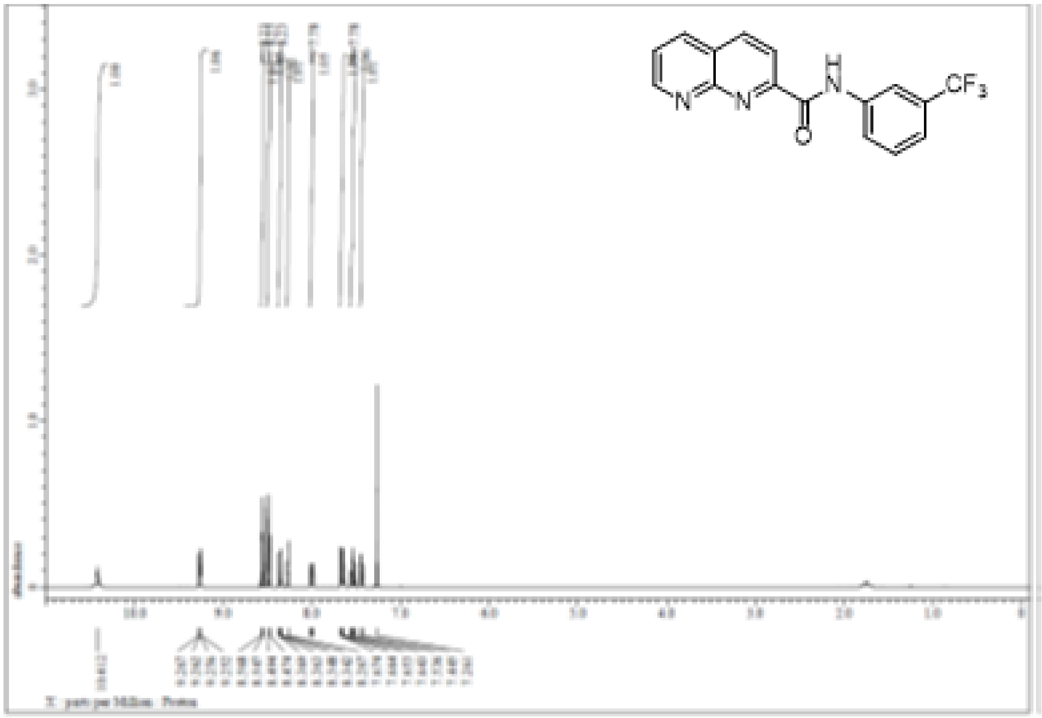

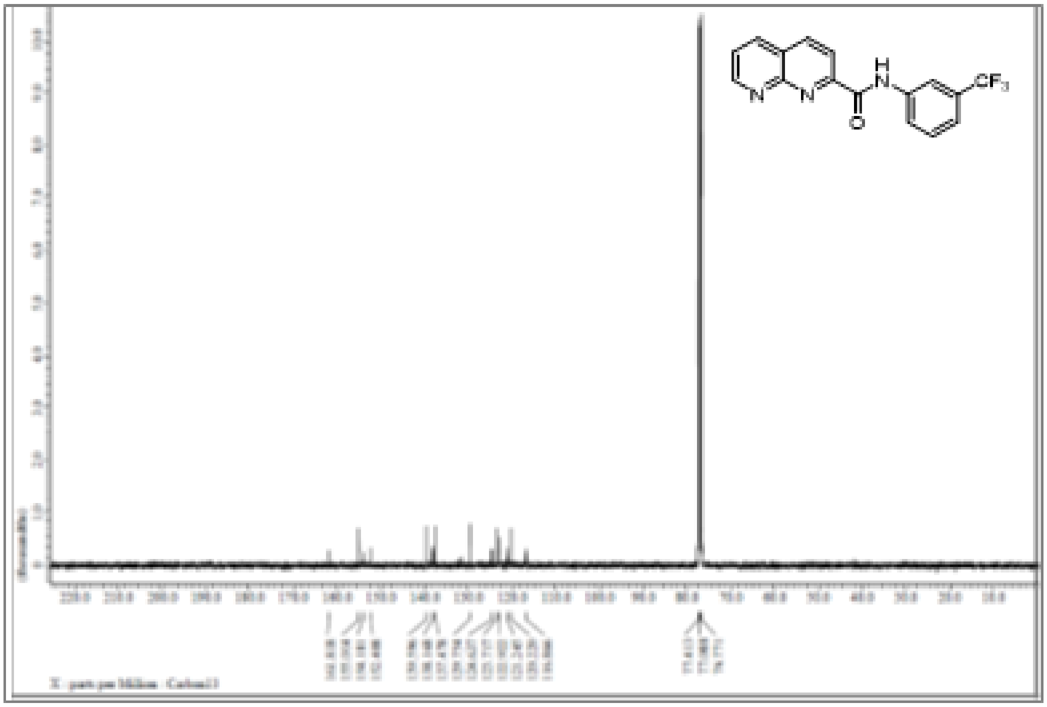

^{1}H and ^{13}C NMR Spectra of HSR2109

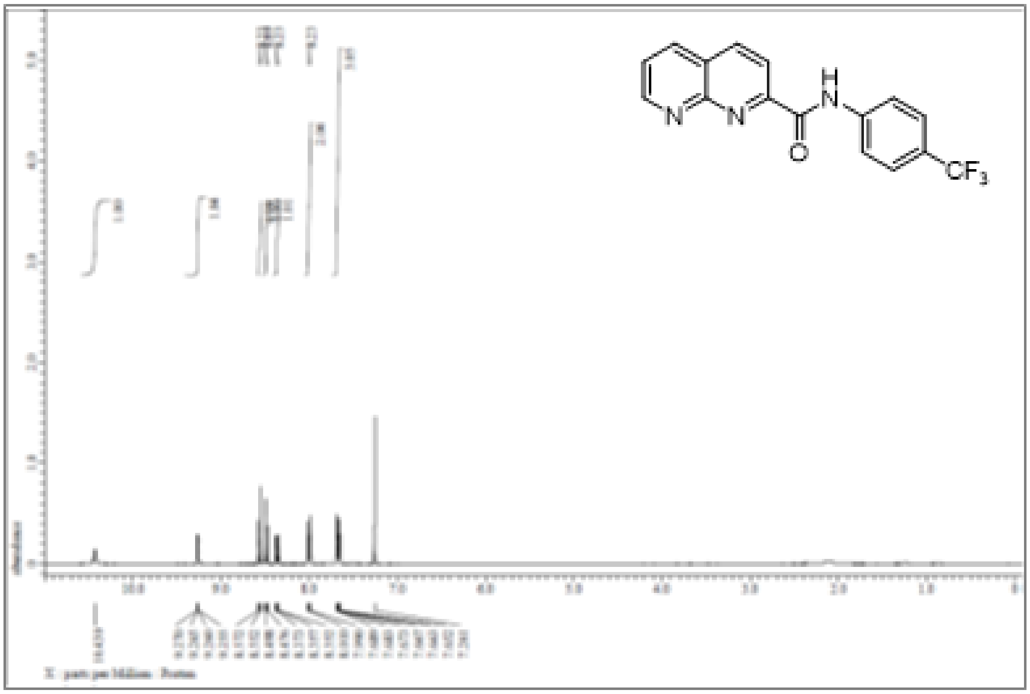

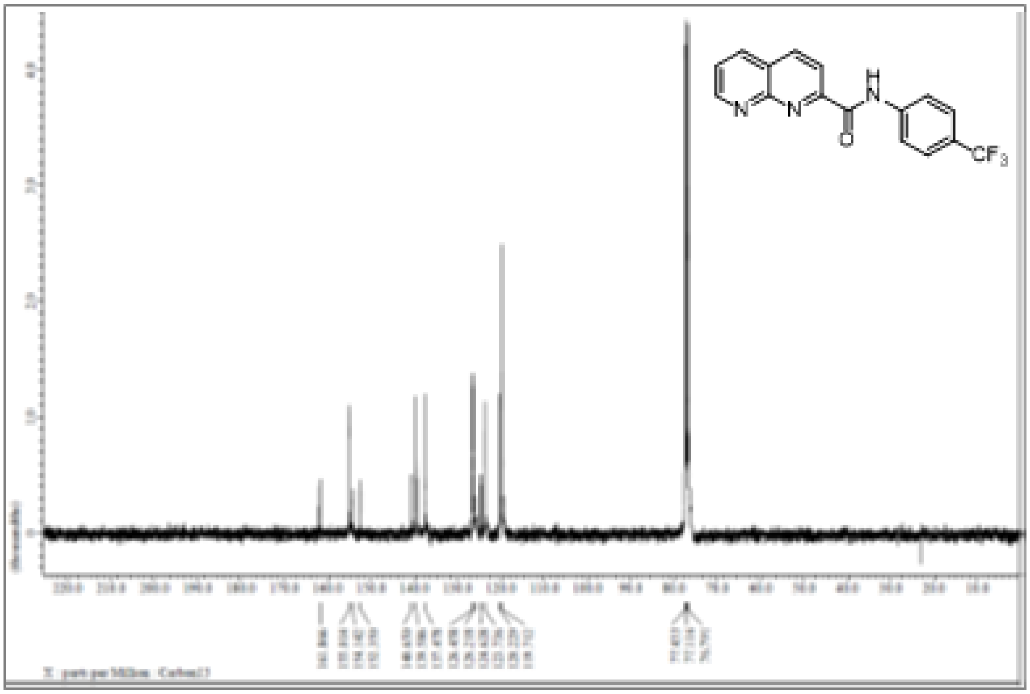

^{1}H and ^{13}C NMR Spectra of HSR2110

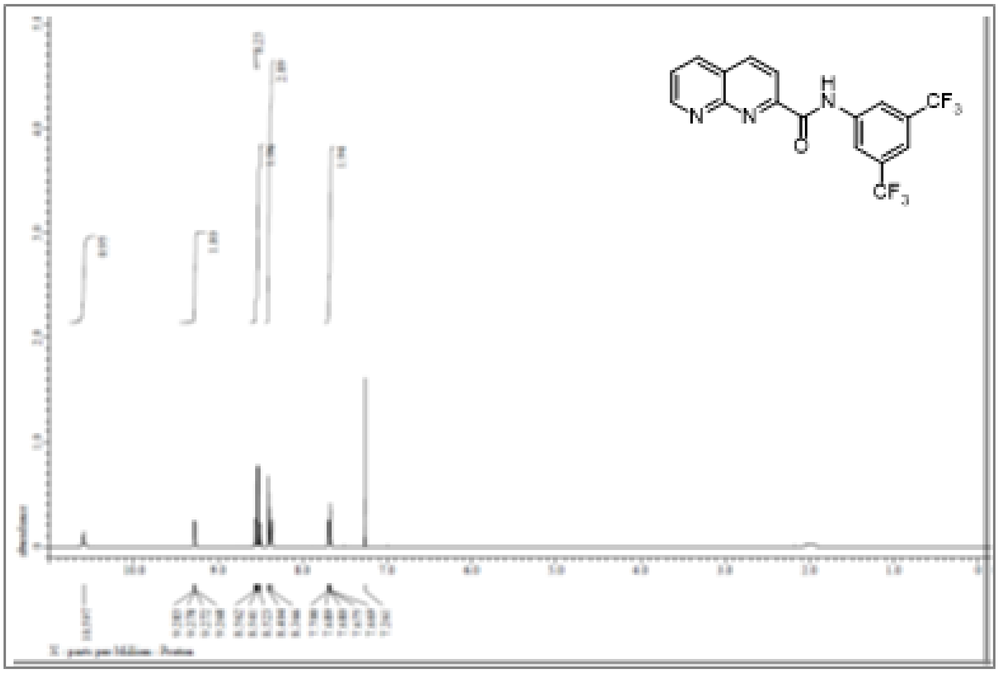

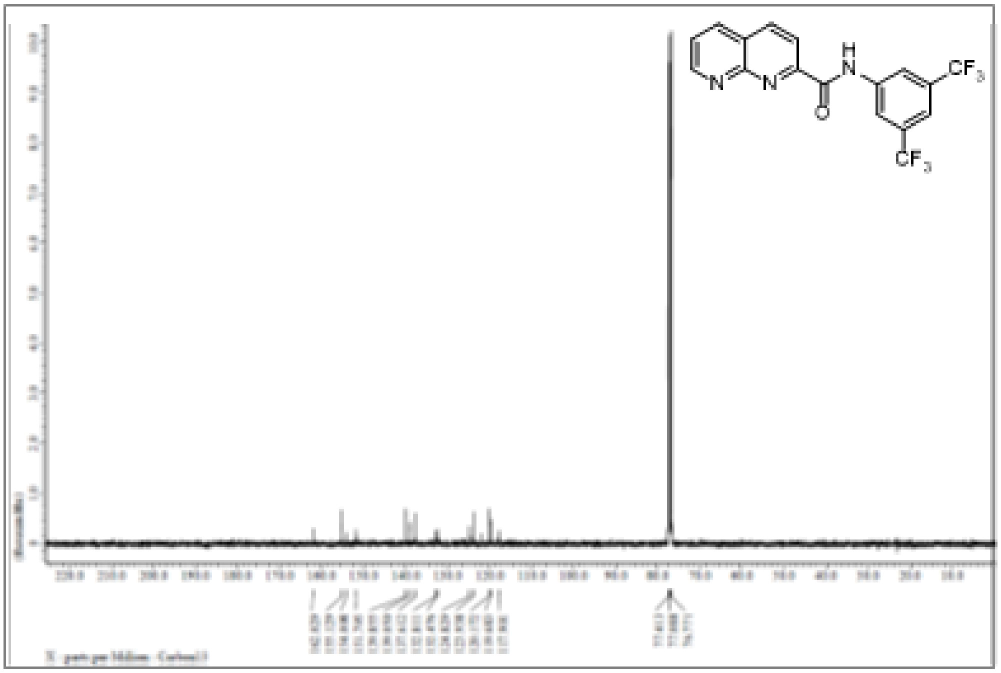

^{1}H and ^{13}C NMR Spectra of HSR2111

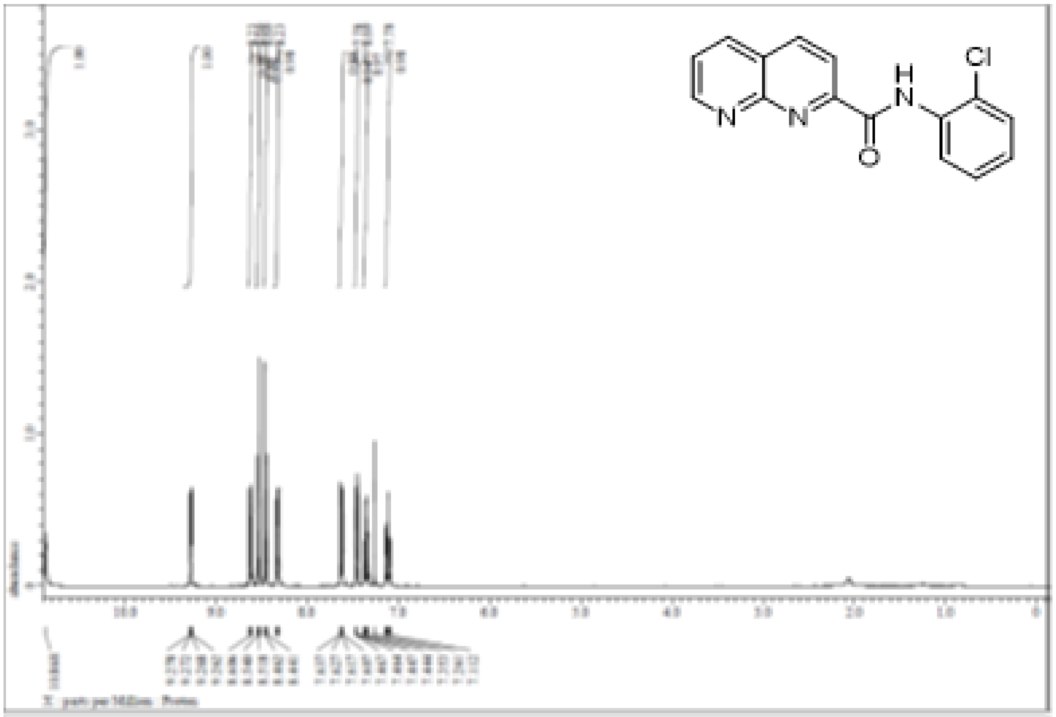

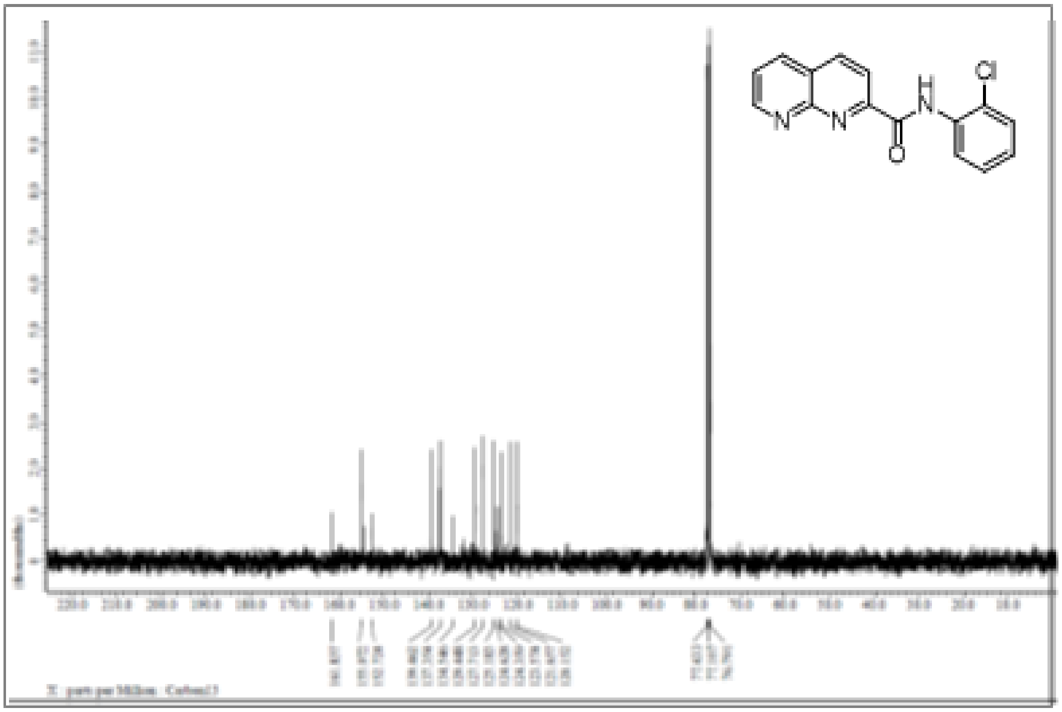

^{1}H and ^{13}C NMR Spectra of HSR2112

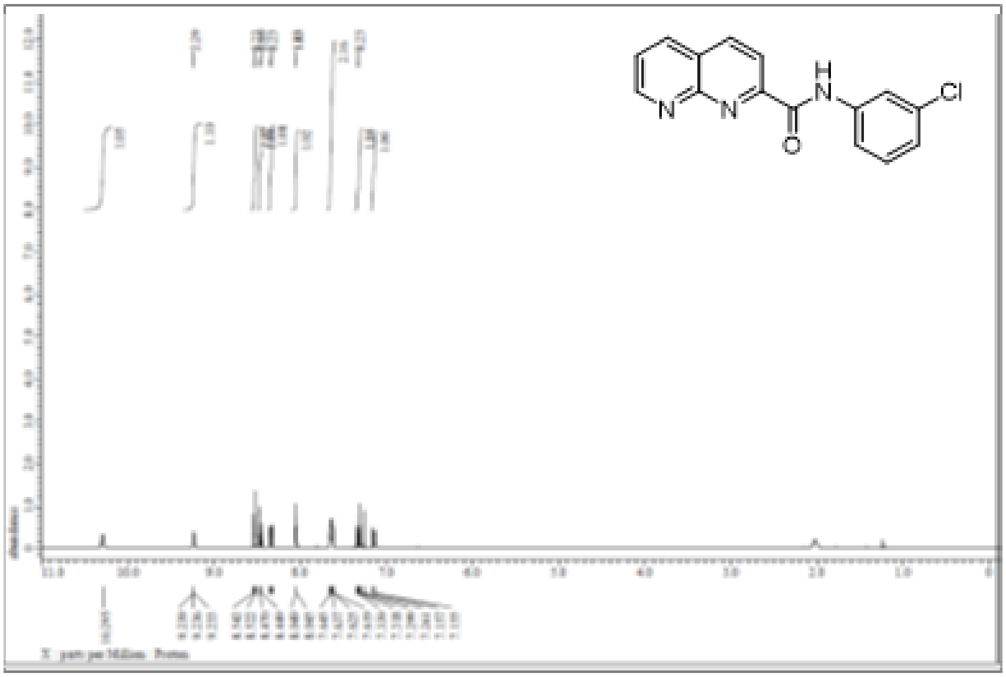

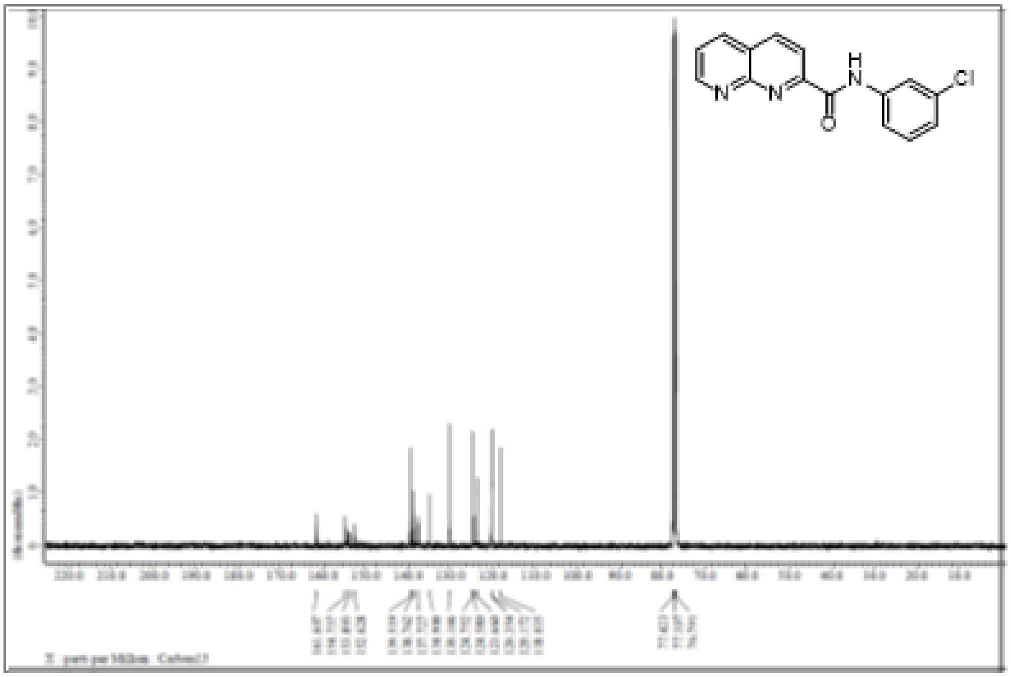

^{1}H and ^{13}C NMR Spectra of HSR2113

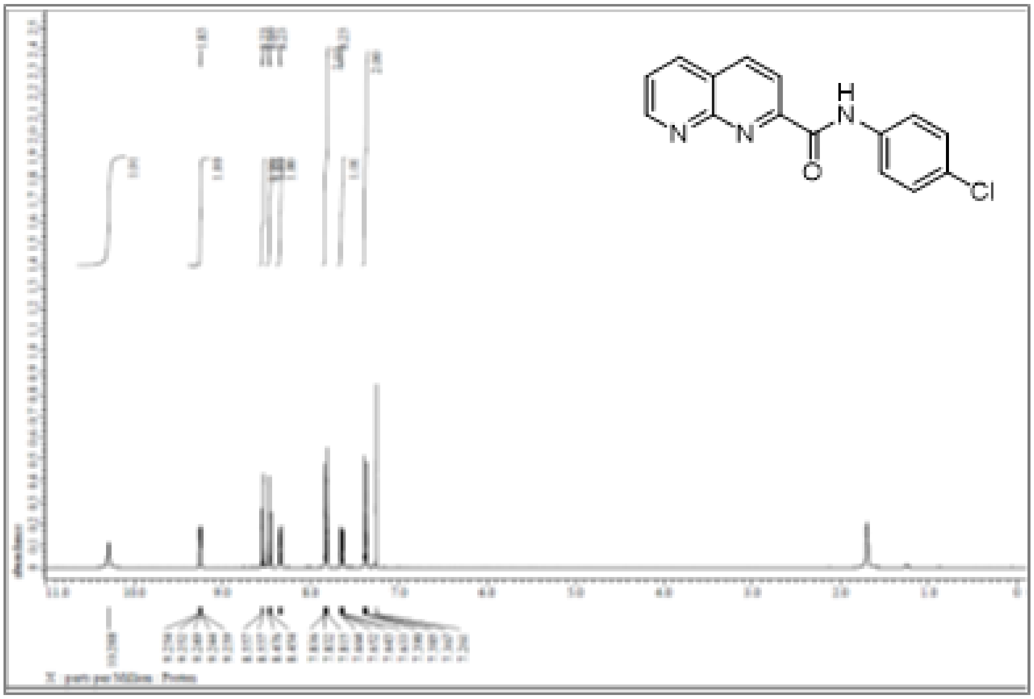

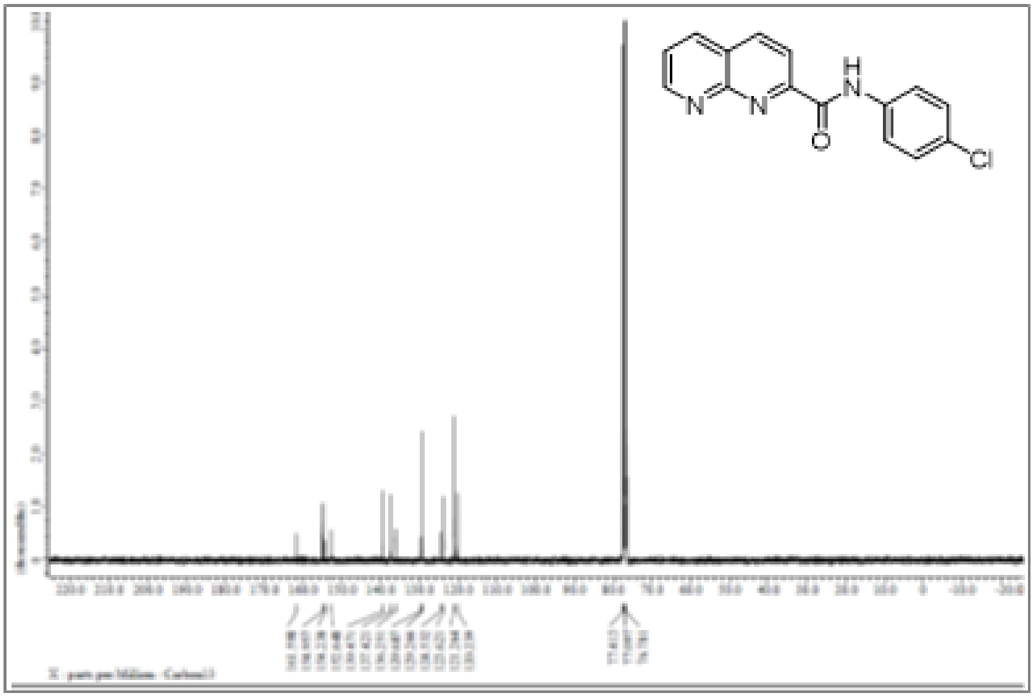

Supplement: Supplementary file 1 [file ijms-22-02527-s001.pdf]
